# Advances in Active Materials of Enzymatic Electrochemical Sensors for Detecting Organophosphorus Pesticides

**DOI:** 10.3390/molecules31040717

**Published:** 2026-02-19

**Authors:** Sijie Ma, Zihang Chen, Fuxiong Yang, Ting Yao, Suo Wang, Yi Yu, Liangbin Xiong, Xiaodong Hong, Guangjin Wang

**Affiliations:** 1 School of Materials and Energy, Foshan University, Foshan 528000, China; 15899654506@163.com (S.M.); 15729508936@163.com (Z.C.); 15207853481@163.com (F.Y.); 18381662518@163.com (T.Y.); 2 The Fifth Electronic Research Institute of the Ministry of Industry and Information Technology, Guangzhou 510610, China; 3 Hubei Key Laboratory of Advanced Technology for Automotive Components, Wuhan University of Technology, Wuhan 430070, China; michael.yy2024@gmail.com; 4 School of Optoelectronic Engineering, Guangdong Polytechnic Normal University, Guangzhou 510665, China; xiongliangbin@gpnu.edu.cn

**Keywords:** organophosphorus pesticides, acetylcholinesterase, enzymatic electrochemical sensors, active materials

## Abstract

Organophosphorus pesticides (OPs) have been widely employed to increase food production and alleviate the increasingly serious food crisis. However, excessive use of these pesticides has seriously affected human health and even caused death due to significant pesticide residues in food. Therefore, enzymatic electrochemical sensors have been developed to monitor OP residues in food. The electrochemical detection performance of these sensors is determined by the physicochemical properties of electrochemical active materials in their active layers. The definition and classification of OPs are first introduced in this review, then the components of enzymatic electrochemical sensors, including electrodes, electrochemical active layer and bioactive enzyme layer, are analyzed in detail. Furthermore, this review emphatically discusses the recent development of enzymatic electrochemical sensors based on various electrochemical active materials: carbon-based, polymer-based, metal-based, metallic compound-based, metal organic framework-based and covalent organic framework-based materials. Finally, probable research directions for developing enzymatic electrochemical sensors with high sensitivity, excellent stability and good reproducibility are outlined to accelerate rapid, effective and low-cost on-site detection OPs in food. This review is expected to provide inspiration for the design and preparation of the high-performance enzymatic electrochemical sensors.

## 1. Introduction

Over the past twenty years, the population of the world has increased by 1.6 billion, which has caused a serious global food crisis [1]. For relieving this problem, pesticides have been employed to eliminate weeds and pests and thus increase food production. According to the data from the China Pesticide Information Network [2], the worldwide total pesticide usage reached about 4 million tons in 2023, which was 2% lower than last year and 14% more than a decade ago. During the same period, the total pesticide usage of China was about 230 thousand tons. Among these used pesticides, the usage amount of organophosphorus pesticides (OPs) has accounted for more than 30% of total pesticide usage due to their low cost, degradability and effectiveness against various weeds and pests [3]. Unfortunately, only a small fraction of OPs is used to eliminate weeds and pests, and majority enters soil and drinking water, thus resulting in food pollution. It is well known that ingesting food containing residual OPs can cause poisoning or even death in humans [4]. Consequently, developing accurate, rapid and sensitive detection technologies to monitor OPs residual in food is of great importance to ensuring human safety.

At present, the most common detection technologies for OP residues are chromatographic methods [5,6], including gas chromatography, gas chromatography–mass spectrometry, liquid chromatography, liquid chromatography–mass spectrometry and high-performance liquid chromatography coupled with ultraviolet detector. These approaches exhibit ultrahigh accuracy, excellent sensitivity and good selectivity for detecting OPs, but they require expensive professional instruments and well-trained technicians. Furthermore, their operation processes are considerably complicated, and the pretreatment of their detection samples takes a long time. Most importantly, these methods are unsuitable for on-site detection of OP residues and only work in laboratories. Therefore, it is urgent to develop accurate, rapid, stable, reliable and simple operational detection technologies for achieving highly sensitive and selective on-site detection of OP residues in food.

Due to their advantages in miniaturization, rapid response and suitability for on-site detection, sensor technologies such as electrochemiluminescence [7], colorimetric [8], fluorescent [9] and electrochemical [10] sensors have been widely employed to detect OP residues in food. Among them, electrochemical sensing technologies including enzymatic/nonenzymatic electrochemical sensors exhibit low cost, simple operation, fast detection, high reliability, good repeatability and selectivity, thus attracting much attention in OP residue detection [11]. Compared with non-enzymatic electrochemical sensors, enzymatic electrochemical sensors are able to simultaneously detect multiple OPs. For this reason, many efforts have been devoted to developing high-performance enzymatic electrochemical sensors. As we all know, the detection performance of enzymatic electrochemical sensors is directly determined by the physicochemical properties of active materials in their electrochemical active layers [12]. Accordingly, the development of active materials is significant for the construction of high-performance enzymatic electrochemical sensors. After continued and unremitting efforts by researchers, studies on active materials for enzymatic electrochemical sensors have made significant process. Therefore, it is significant to summarize these advances to provide inspiration for the design and preparation of the high-performance enzymatic electrochemical sensors.

Recently, Zeng et al. [13] summarized the research progress in the development of acetylcholinesterase-based sensors for detection of OPs in food and environment samples, focusing on the immobilization strategies of acetylcholinesterase (AChE) and the development progress of AChE-based optical and capillary electrophoresis sensing detection technologies. However, the systematic summaries on the recent advances in active materials for high-performance enzymatic electrochemical sensors are still lacking. Hence, we will initially discuss the definition and classification of OPs, then detail the components of enzymatic electrochemical sensors, including electrodes, electrochemical active layer and bioactive enzyme layer. The recent development of enzymatic electrochemical sensors based on various electrochemical active materials such as carbon-based, polymer-based, metal-based, metallic compound-based, metal organic framework (MOF)-based and covalent organic framework (COF)-based materials will be emphatically summarized. Finally, probable research directions for developing enzymatic electrochemical sensors with high sensitivity, excellent stability and good reproducibility are outlined to accelerate rapid, effective and low-cost on-site detection of OPs in food.

## 2. The Definition and Classification of OPs

**Figure 1:**
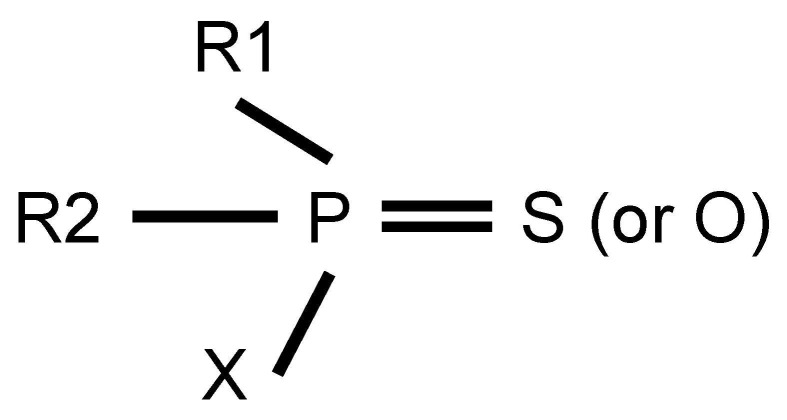
The general molecular formula of OPs.

OPs are one of the important pesticides for controlling weeds and pests [3]. Their active agents are organic phosphorus compounds, where a phosphorus atom binds to oxygen, nitrogen, sulfur, halogen atoms and organic groups such as alkoxy, alkyl, aryl and amino groups via covalent bonds. The general molecular formula of OPs is shown in Figure 1. R1 and R2 represent organic groups (alkoxy, alkyl, aryl and amino, etc.), while X denotes leaving groups. These leaving groups are prone to hydrolysis by hydrolases or substitute through phosphorylation of AChE. Until now, OPs have been divided into different types based on the different classified criteria. In terms of chemical structure and application [14], 71 common OPs are used for eradicating weeds, fungi and pests. Simultaneously, according to toxicological mechanism [15], OPs can be classified into 13 subtypes, including phosphates, phosphinates, phosphonofluoridates, phosphonates, phosphorothioates, phosphoramidothioates, phosphorodithioates, phosphoramidates, s-substituted phos-phorothioates, phosphonothioates, phosphorofluoridates, s-substituted phosphonothioates and s-substituted phosphoramidothioates. In addition, based on toxicity level, OPs can be divided into low, middle and high toxicity [16].

Based on data from the National Center for Biotechnology Information of National Library of Medicine [17], Table 1 summarizes the chemical name (based on International Union of Pure and Applied Chemistry, IUPAC), molecular formula and physicochemical properties of OPs which have been reported in Section 4 of this paper. As shown in Table 1, most OPs are hydrolysis in alkaline condition, but trichlorfon is rapid isomerization in alkaline condition. The majority of OPs are stable in neutral condition, and a small fraction are stable in weakly acidic condition. As such, the application and detection of OPs are recommended under neutral or weakly acidic conditions. On the one hand, almost all OPs are hardly volatile, but dichlorvos is a highly volatile and toxic at ambient temperature and pressure, requiring careful handling during spraying. On the other hand, most OPs are photosensitive and thermosensitive, necessitating storage in dark and room-temperature conditions. It is worth noting that phoxim with high photosensitivity is suitable for underground pest control. On the contrary, acephate, glyphosate, pirimiphos methyl, trichlorfon and profenofos display high photostability and thermostability, making them problematic due to long-term persistence in the food chain. For solubility, all OPs are soluble in organic solvent, and some are slightly dissolved in water, nonpolar solutions, aliphatic hydrocarbons or mineral oils. A few OPs such as acephate, glyphosate, trichlorfon, methamidophos, methomyl, and monocrotophos are extremely soluble in water. Therefore, based on toxicity, stability and solubility, acephate, glyphosate and trichlorfon may be the ideal OP models for evaluating the detection performance of enzymatic electrochemical sensors.

## 3. The Construction of Enzymatic Electrochemical Sensors

**Figure 2:**
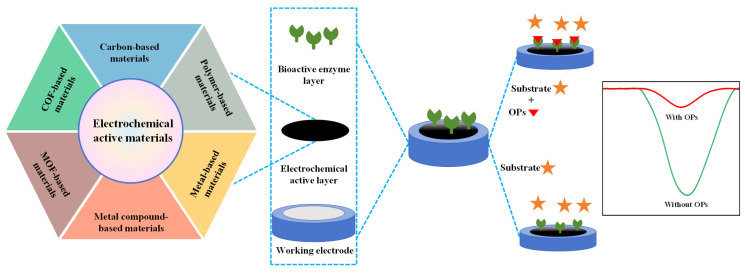
The construction of enzymatic electrochemical sensors and their detection mechanism.

As shown in Figure 2, typical enzymatic electrochemical sensors consist of three core components: (1) electrodes include a reference, auxiliary and working electrode in traditional three-electrode system; (2) electrochemical active layer contains non-active site blocking agents, adhesion agents and electrochemical active materials; and (3) bioactive enzyme layer refers to AChE, butyrylcholinesterase and organophosphorus hydrolase layer. Each component will be discussed in the following section.

### 3.1. Electrodes

Enzymatic electrochemical sensors based on traditional three-electrode systems contain reference, auxiliary and working electrodes [18]. Therein, the reference electrode is able to provide the stable, reversible and reproducible reference potential for calibrating the applied potential of working electrode. The ideal reference electrode requires stable potential, low polarization and good compatible with tested system. The auxiliary electrode forms a close current circuit with the working electrode for conducting the reverse current of the working electrode and ensuring the stable and continuous process of the target electrochemical reaction on the working electrode. The ideal auxiliary electrode requires good conductivity, high stability and chemical inertness. The working electrode is capable of transforming the charge change in the electrochemical reaction of the target OPs into detectable current, potential and resistance signal. The electrochemical properties of the electrochemical active material modified working electrode directly determine the detection performances of three-electrode enzymatic electrochemical sensors. The requirements for an ideal working electrode are the same as an auxiliary electrode, that is, high stability, excellent conductivity and chemical inertness. According to the summarized literature in Section 4 of this paper, the common working electrodes for the constructure of enzymatic electrochemical sensors contain glass carbon electrode (GCE), scree-printed electrode (SPE), boron-doped diamond electrode (BDDE), indium tin oxide electrode (ITOE), fluorine-doped tin oxide electrode (FTOE) and gold electrode (GE).

### 3.2. Electrochemical Active Layer

The electrochemical active layer on the working electrode is the most crucial functional layer in enzymatic electrochemical sensors which connects to the working electrode and the bioactive enzyme layer and gives assistance to the generation, transmission and amplification of detection signals. As such, the detection performances of electrochemical sensors for target OPs are directly determined by the electrical performances of the electrochemical active layer. The main functions of the electrochemical active layer are providing catalytic active sites for the electrochemical reaction of target OPs, accelerating the charge transfer between the working electrode and the bioactive enzyme layer and boosting the fixation of bioactive enzymes. Accordingly, the rational optimization of the electrochemical active layer is significant for providing enzymatic electrochemical sensors with distinguished electrochemical performances for the detection of target OPs.

It is well known that the electrochemical active layer in enzymatic electrochemical sensors is made up of non-active site blocking agents, adhesion agents and electrochemical active materials. According to the summarized literature in Section 4 of this paper, the common non-active site blocking agent is bovine serum albumin (BSA), which has abundant amino and carboxyl groups [19]. Additionally, the most common adhesion agents refer to chitosan (Chi), glutaraldehyde and nafion. Therein, Chi is the deacetylation product of chitin, which has abundant amino and carboxyl groups in its molecular chain [20]. These functional groups contribute to the incorporation with bioactive enzymes by weak physicochemical interactions such as hydrogen bond or electrostatic effect, achieving the mild fixation of bioactive enzymes and avoiding the inactivation of bioactive enzymes caused by very slight physical adsorption and strong chemical crosslinking. What is more important is that the hydrophilic microenvironment from Chi is conducive to maintaining the spatial conformation of bioactive enzymes and enhancing the catalytic activity and storage stability of bioactive enzymes. Additionally, there is an aldehyde group in each end of glutaraldehyde molecular chain. Due to the strong chemical interaction between the aldehyde group of glutaraldehyde and the amino group of bioactive enzymes, the bioactive enzymes are strongly anchored into the three-dimensional network of the electrochemical active layer by glutaraldehyde [21]. Their strong chemical interaction is likely to cause the change in the spatial conformation of bioactive enzymes, which in turn gives rise to the inaction of bioactive enzymes. Unlike with glutaraldehyde, the nafion composing the polytetrafluoroethylene framework and sulfonic acid group immobilizes bioactive enzymes on the electrochemical active layer through slight physical adsorption [22]. This very slight physical adsorption may cause the loss of bioactive enzymes on the electrochemical active layer, thereby resulting in the reduction in detection performance of enzymatic electrochemical sensors.

The active materials in the electrochemical active layer are the core functional materials of enzymatic electrochemical sensors. The active materials, which are coated on the working electrode surface, directly participate the electrochemical reaction of target OPs and promote the amplification of detectable response signals. To prepare high-performance enzymatic electrochemical sensors, it is necessary to exploit the electrochemical active materials with excellent conductivity, outstanding electrochemical catalytic activity, high specific surface area, controllable porous structure, considerable electro-/chemical stability, strong anti-interference ability and good biocompatibility. Consequently, various electrochemical active materials such as carbon-, polymer-, metal-, metallic compound-, metal organic framework- and covalent organic framework-based materials have attracted much attention in the development of high-performance enzymatic electrochemical sensors. After the continuous efforts of researchers, studies on these aforementioned electrochemical active materials for the development of high-performance enzymatic electrochemical sensors have made many breakthroughs which will be emphatically discussed in Section 4 of this paper.

### 3.3. Bioactive Enzyme Layer

The bioactive enzyme layer referring to AChE, butyrylcholinesterase and the organophosphorus hydrolase layer is the signal recognition element in enzymatic electrochemical sensors. It is well known that AChE is one of the most important serine hydrolases extracted from various organisms or tissues such as marine organisms, insects, plants and human serum [23]. The primary structure of AChE consists of over 500 amino acid residues, and its catalytic domain is the serine (Ser203)–histidine (His447)–glutamic acid (Glu334) triplet. In the secondary structure of AChE, α/β hydrolase folding is the core architecture of the catalytic domain; in the tertiary structure, the catalytic domain folds into a spherical conformation with a diameter of about 45 Å. The quaternary structure of AChE is assembled from catalytic and anchoring subunits. The hydroxyl groups of Ser203 can specifically bind with the phosphoric groups of OPs through robust covalent bonds, forming stable phosphorylated enzyme complexes and causing the irreversible inactivation of AChE [24]. Analogous to AChE, butyrylcholinesterase also belongs to the cholinesterase family but is mainly derived from animal serum. Their structural difference lies in the number of amino acid residues and the composition of the active triplet [25], yet they interact with OPs in the same manner. Therefore, AChE and butyrylcholinesterase are employed as signal recognition elements to build inhibited enzymatic electrochemical sensors for OP detection. For inhibited enzymatic electrochemical sensors, both bioactive enzymes could catalyze the hydrolysis of acetylsalicycholine to produce thiocholine, which is oxidized on the electrochemical active layer to generate detectable response current. In the presence of OPs in the electrolyte solution, the response current intensity decreased due to the catalytic activity of enzyme inhibition by OPs. The electrochemical detection performance of these sensors can be evaluated based on the linear relationship between the response current intensity of sensors and the OP concentration.

Unlike the inhibitory effect of AChE and butyrylcholinesterase, organophosphorus hydrolase extracted from pseudomonas, xanthobacter or *Escherichia coli* (colon bacillus) can directly catalyze the cleavage of P-O/S bonds in OPs, producing p-nitrophenol. This product is oxidized on the electrochemical active layer of catalytic enzymatic electrochemical sensors to generate a detectable response current [26]. The electrochemical detection performance of these sensors can be evaluated on the basis of the linear relationship between the response current of sensors and the OP concentration. Furthermore, organophosphorus hydrolase can be reused and is suitable for continuous online monitoring because of its stable catalytic activity. However, compared to AChE and butyrylcholinesterase, organophosphorus hydrolase has several notable limitations, including high cost, poor stability, short half-life, low reproducibility, complex purification, insufficient broad-spectrum detection of OPs and significant loss of catalytic activity after immobilization [27,28,29]. For this reason, the following section will summarize and review the research progress of electrochemical active materials in inhibited enzymatic electrochemical sensors.

## 4. Electrochemical Active Materials

Due to their excellent electrical conductivity, superior catalytic activity, high specific surface area, remarkable electro-/chemical stability, strong anti-interference capability and outstanding biocompatibility, carbon-based, polymer-based, metal-based, metal compound-based, MOF-based and COF-based materials have attracted extensive attention as active materials for constructing the electrochemical active layer in the development of high-performance enzymatic electrochemical sensors. Therefore, in this section, we comprehensively summarize and review the detection performance of enzymatic electrochemical sensors based on these active materials.

### 4.1. Carbon-Based Materials

**Figure 3:**
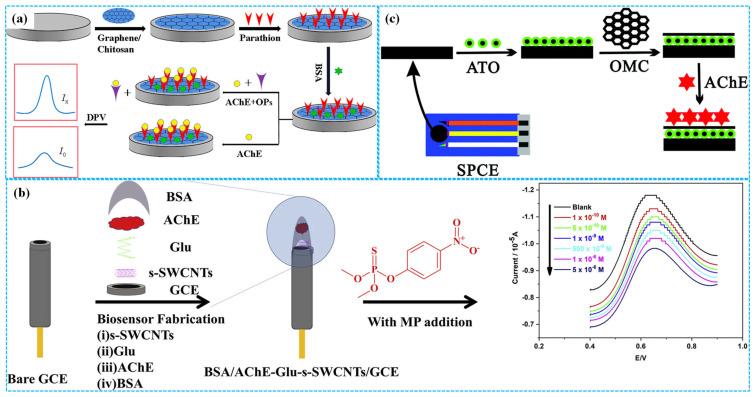
The preparation of Gra−Chi−based ((**a**), reprinted with permission from Ref. [31], Copyright 2021, Elsevier), SWCNT−based ((**b**), reprinted with permission from Ref. [32], Copyright 2019, Elsevier) and ATO−Chi−MC−based ((**c**), reprinted with permission from Ref. [34], Copyright 2019, Royal Society of Chemistry) enzymatic electrochemical sensors.

Owing to their excellent electrical conductivity, favorable chemical and electrochemical stability, high specific surface area, robust mechanical properties, versatile surface functionalization and low density [30], carbon-based materials such as graphene (Gra), carbon nanotubes (CNTs), carbon black (CB) and porous carbon (PC) have been widely investigated for the preparation of enzymatic electrochemical sensors. For instance, Xie et al. [31] coated parathion and BSA on the surface of GCE modified with Gra and Chi composites (Gra-Chi) ink. They exploited the phosphorylation competition of AChE between the parathion immobilized on the modified electrode and target organophosphorus pesticides (OPs) in the electrolyte solution to achieve OP detection, as illustrated in Figure 3a. Under optimized electrochemical conditions, differential pulse voltammetry (DPV) analysis revealed that the peak response current of the modified electrode for chlorpyrifos, dichlorvos, methamidophos, dimethoate, phoxim, parathion, omethoate, pirimiphos methyl, diazinon, fenthion, trichlorfon and methomyl increased with increasing analyte concentration. All target OPs exhibited a same linear concentration range of 1–1500 ng⋅mL^−1^ and limit of detection (LOD) spanning from 0.012 to 0.23 ng⋅mL^−1^.

To eliminate the adverse effects of crosslinking agents on the detection properties of enzymatic electrochemical sensors and improve their electron transfer efficiency, Thakkar et al. [29] first modified GCE with carboxylic group-decorated multiwalled carbon nanotubes (MWCNTs). Subsequently, AChE was fixed on the modified electrode by the amidation reaction between carboxylic groups and amine groups of AChE. Under optimal reaction conditions, cyclic voltammetry (CV) analysis demonstrated that the peak response current of the modified electrode decreased with the increase in the concentration of paraoxon, with a linear concentration range of 10–50 nM and an LOD of 0.1 nM. The modified electrodes exhibited high replication, stability and reactivation for detecting paraoxon. In contrast, Sundramoorthy et al. [32] used glutaraldehyde as a crosslinker to immobilize AChE on single-walled carbon nanotubes’ (SWCNTs) modified GCE surface, preparing enzymatic electrochemical sensors for detection of methyl parathion, as shown in Figure 3b. Under optimized detection conditions, square wave voltammetry (SWV) analysis showed that the response current peak decreased with the increase in the concentration of methyl parathion. The linear concentration range was 1 × 10^−10^–5 × 10^−6^ M and the LOD was 3.75 × 10^−11^ M. The sensors displayed high stability, excellent selectivity and good reproducibility. Furthermore, Han et al. [33] prepared porous Gra oxide (PGO) via a traditional hydrothermal approach and fixed AChE on PGO-modified GCE to build enzymatic electrochemical sensors for detecting paraoxon. Under optimal electrochemical conditions, DPV analysis showed that the inhibition rate of the modified electrode increased with the increase in the concentration of paraoxon. The linear concentration range was 10–45 ng mL^−1^ and the LOD was 1.58 ng mL^−1^. The recovery for paraoxon was 96.9–100.4% in vegetables samples.

In addition, Guo et al. [34] reported the use of antimony tin oxide, Chi and mesoporous carbon composites (ATO-Chi-MC) to decorate SPE, followed by AChE immobilization for the detection of chlorpyrifos and methamidophos, as shown in Figure 3c. Under optimal reaction conditions, DPV analysis confirmed that the sensors exhibited superior electrochemical detection performances, good storage stability and excellent repeatability. In the chlorpyrifos concentration range of 0.01–105 μg L^−1^, the response current peak of sensors decreased with the increase in its concentration with a corresponding LOD of 0.01 μg L^−1^. In the same linear concentration range, the LOD of sensors for detecting methamidophos was 1 μg L^−1^. More importantly, enzymatic electrochemical sensors can also be used for simultaneously detecting carbamate pesticides and OPs. For example, Zergioti et al. [35] immobilized AChE on the surface of SPE modified with CB to prepare enzymatic electrochemical sensors for the simultaneous detection of chlorpyrifos and carbofuran. Under optimal conditions, the sensors displayed a linear relationship between their response current peak and the concentration of both pesticides. The linear concentration range was 0.7 × 10^−9^–1.4 × 10^−8^ mol L^−1^ for chlorpyrifos (LOD = 6 × 10^−10^ mol L^−1^) and 1.1 × 10^−9^–2.3 × 10^−8^ mol L^−1^ for carbofuran (LOD = 4 × 10^−10^ mol L^−1^).

Moreover, Wei et al. [36] modified BDDE with nitrogen-doped PC (N-PC, pore diameter of 150 nm), which was prepared by a hard template method using 1-butyl-3-methylimidazolium dicyanamide ionic liquid as precursors and silica spheres as a template. AChE was then coated on the modified electrode to construct enzymatic electrochemical sensors for detection of OPs such as dichlorvos and fenitrothion. Under optimal electrochemical conditions, chronoamperometry analysis showed that the inhibition rate of the modified electrode increased with the increase in the concentration of OPs, with the same linear concentration range in 10^−10^–10^−5^ g L^−1^ for both OPs. The LOD was 1.50 pg·L^−1^ for dichlorvos and 4.42 pg·L^−1^ for fenitrothion. The modified electrodes exhibited excellent repeatability reproducibility, high stability, good selectivity and remarkable recoveries for both OPs in real samples. The superior electrochemical performance of the modified electrodes was attributed to the high conductivity of N-PC (~79 Ω) which is smaller than that of bare BDDE (~365 Ω) and PC-modified BDDE (~199 Ω).

Although carbon-based enzymatic electrochemical sensors have demonstrated outstanding performance for detecting various OPs such as ultralow sensitivity, good stability and considerable recovery in real samples, their practical applications are hindered by their intrinsic drawbacks, including insufficient dispersibility, weak interaction with bioactive enzymes, limited selectivity and low reproducibility. Therefore, strategies such as surface modification, heteroatom doping and composite formation with crosslinking agents can be employed to improve the detection performance of carbon-based enzymatic electrochemical sensors.

### 4.2. Polymer-Based Materials

Benefiting from their excellent conductivity derived from π conjugated structure, outstanding biocompatibility, favorable porosity, considerable mechanical stability, remarkable chemical stability and high specific surface area [37], polymer-based materials such as polyaniline (PANI), polyimide (PI), polypyrrole (PPY), poly 3,4-ethylene dioxy thiophene (PEDOT), polystyrene sulfonate (PSS) and 4, 7-di (furan-2-yl) benzo [1,2,5] thiadiazole (FBThF), are widely employed as bioactive enzyme immobilization carriers to the fabrication of enzymatic electrochemical sensors for detecting OPs.

**Figure 4:**
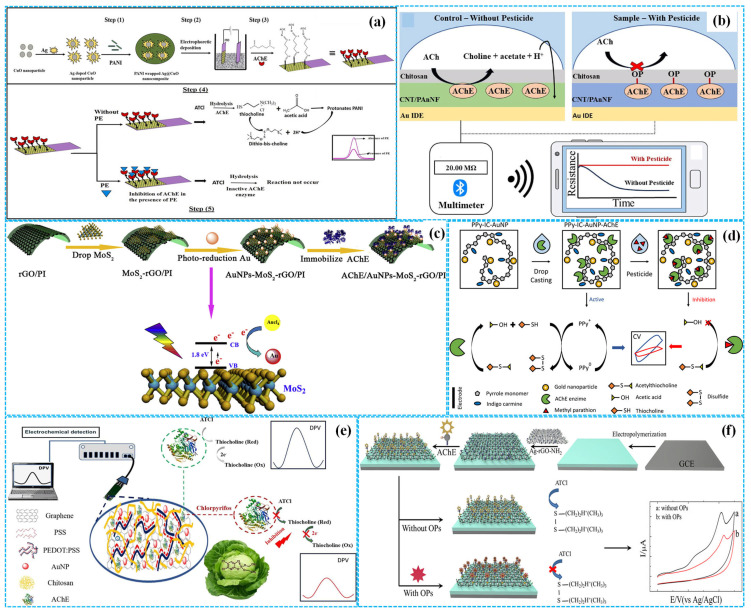
The preparation of Ag−CuO−PANI−based ((**a**), reprinted with permission from Ref. [38], Copyright 2023, Elsevier), Chi−PANI NFs−CNT−based ((**b**), reprinted with permission from Ref. [40], Copyright 2023, Elsevier), Au NPs−MoS_2_−rGO−PI−based ((**c**), reprinted with permission from Ref. [41], Copyright 2020, Elsevier), Au NPs−PPY−based ((**d**), reprinted with permission from Ref. [43], Copyright 2025, Elsevier), Au NPs−Gra−PSS−PDOT−based ((**e**), reprinted with permission from Ref. [45], Copyright 2023, Elsevier) and Ag NPs−rGO−FBThF−based ((**f**), reprinted with permission from Ref. [46], Copyright 2019, Elsevier) enzymatic electrochemical sensors.

As shown in Figure 4a, Kumar et al. [38] modified ITOE with Ag-CuO nanoparticles and PANI composites (Ag-CuO-PANI) with an electrodeposited approach, followed by glutaraldehyde-mediated AChE immobilization to prepare enzymatic electrochemical sensors. Under optimal reaction conditions, DPV analysis revealed the peak response current decreased with the increase in with the concentration of paraoxon, with a linear concentration range of 5–100 pM, a sensitivity of 0.5536 μA (pM)^−1^ cm^−2^ and an LOD of 11.35 pM. The recoveries were 92–111% for paraoxon in banana, tomato and soil samples. The excellent electrochemical performance was related to the uniform distribution of catalytic active constituents within the PANI matrix. Based on the unique conduction characteristics of doping/dedoping PANI [39], Wang et al. [40] developed enzymatic resistive sensors by coating composition films consisting of Chi, AChE, PANI nanofibers and CNTs (Chi-PANI NFs-CNTs) on GE, as illustrated in Figure 4b. These sensors were combined with smartphones for on-site detection of paraoxon. Under optimized conditions, a linear relationship was observed between the response resistance of portable sensors and the concentration of paraoxon-methyl, in a concentration range of 1 ppt–100 ppb with an LOD of 0.304 ppt. The portable sensor achieved satisfactory recovery rates, which were comparable to those of traditional mass spectrometry methods for real sample analysis.

Furthermore, Jia et al. [41] used the composites of PI and reduced Gra oxide (rGO) as a flexible working electrode and fabricated enzymatic electrochemical sensors by the photoreduction of Au nanoparticles (Au NPs) and the fixation of monolayer MoS_2_ and AChE (AChE-Au NPs-MoS_2_-rGO-PI), as shown in Figure 4c. Under optimized reaction conditions, DPV analysis demonstrated that the linear relationship between the peak response current of sensors and the concertation of paraoxon existed in the concentration range of 0.005–0.150 μg mL^−1^ with a sensitivity of 4.44 μA (μg mL^−1^)^−1^ and an LOD of 0.0014 μg mL^−1^.

Owing to the promotion of Au NPs for the nucleation of PPY at applied potential [42], Santos et al. [43] integrated Au NPs into indigo carmine doped PPY (Au NPs-IC-PPY) by electrochemical synthesis methods using FTOE as working electrodes. AChE was then immobilized to construct enzymatic electrochemical sensors for detecting methyl parathion, as shown in Figure 4d. Under optimized detection conditions, CV analysis showed that the difference between anodic and cathodic response peak currents of sensors was linearly dependent on the concentration of methyl parathion, in the concentration range of 1.3 × 10^−7^–1.0 × 10^−3^ mol L^−1^, with an LOD of 24 fmol L^−1^. The outstanding electrochemical performance of sensors was ascribed to the synergistic effect between the high biocompatibility of indigo carmine doped PPY chains and the good conductivity of Au NPs. Subsequently, Santos et al. [44] introduced dodecyl sulfate and indigo carmine into the PPY matrix (DS-IC-PPY) to further improve its conductivity, stability and electroactivity. Using the same preparation method, enzymatic electrochemical sensors for detecting carbaryl were constructed. Under optimal reaction conditions, the sensors showed high sensitivity with a linear carbaryl concentration of 0.05–0.25 ng mL^−1^, an LOD of 0.033 ng cm^2^ mL^−1^ and a limit of quantification (QOD) of 0.11 ng cm^2^ mL^−1^.

In addition, Dechtrirat et al. [45] incorporated Gra sheets into PEDOT and PSS mixed matrices, followed by Au NP decoration (Au NPs-Gra-PEDOT-PSS). The prepared composites were inkjet-printed on SPE, and AChE was immobilized to construct enzymatic electrochemical sensors for detecting chlorpyrifos (Figure 4e). Under optimal reaction conditions, DPV analysis indicated the peak response current of sensors decreased with the concentration of chlorpyrifos. The linear concentration range was 0.1–10 nM, and the LOD was 0.07 nM. The excellent electrochemical performance was correlated with the high conductivity of each component in the composites. Moreover, Pan et al. [46] prepared FBThF films on GCE using electro-polymerization, followed by modification with the mixtures of Ag nanoparticles (Ag NPs) and amino group-decorated rGO (Ag NPs-rGO-FBThF) and AChE fixation to fabricate enzymatic electrochemical sensors, as shown in Figure 4f. Under optimal detection conditions, CV analysis showed that the inhibition rate of sensors was directly proportional to the concentration of OPs. The linear concentration range was 0.099–9.9 μg L^−1^ for malathion and 0.0206–2.06 μg L^−1^ for trichlorfon, respectively. Their corresponding LOD was about 0.032 μg L^−1^ and 0.001 μg L^−1^. The sensors displayed remarkable reproducibility, good stability and high recovery for both OPs in real samples.

Enzymatic electrochemical sensors based on polymer-based materials have exhibited excellent electrochemical performances for detecting OPs. However, their detection performance is limited by inherent drawbacks, such as structural degradation under sensor operating conditions (leading to rapid conductivity loss), non-specific interactions with OPs (reducing sensor selectivity), reliance on high-purity monomers and specific dopants for synthesis and uneven physicochemical properties caused by complex preparation processes. Therefore, strategies including composite formation with other inorganic and carbon-based materials, surface functional modification, preparation process optimization and structural design refinement can be applied to improve the detection performance of polymer-based enzymatic electrochemical sensors.

### 4.3. Metal-Based Materials

**Figure 5:**
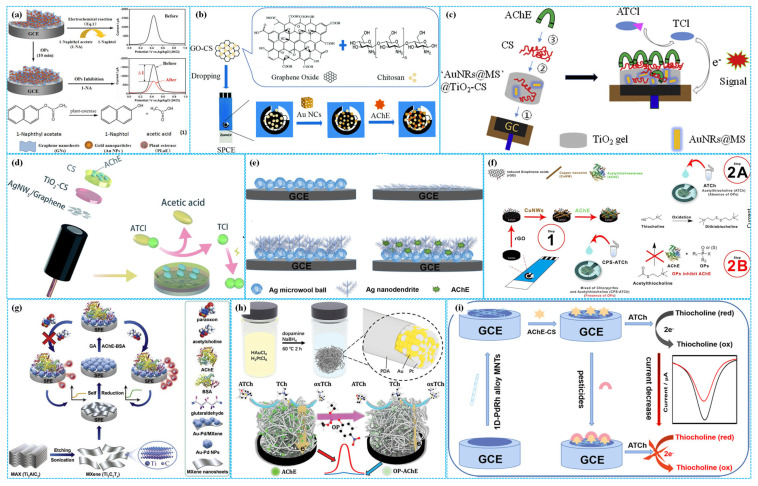
The preparation of Au NPs−PE−Chi−Gra−based ((**a**), reprinted with permission from Ref. [50], Copyright 2015, American Chemical Society), Au NPs−GO−Chi−based ((**b**), reprinted with permission from Ref. [51], Copyright 2019, Royal Society of Chemistry), Au NPs−SiO_2_−TiO_2_−based ((**c**), reprinted with permission from Ref. [52], Copyright 2019, Elsevier), TiO_2_−Chi−Ag NFs−Gra−Chi−based (**d**), 3D Ag TN−based ((**e**), reprinted with permission from Ref. [56], Copyright 2020, Elsevier), Cu NWs−rGO−based ((**f**), reprinted with permission from Ref. [57], Copyright 2024, Elsevier), AuPd NPs−MXene−based ((**g**), reprinted with permission from Ref. [59], Copyright 2020, Elsevier), PD−AuPt−based ((**h**), reprinted with permission from Ref. [60], Copyright 2019, John Wiley and Sons) and 1D PdRh NT−based ((**i**), reprinted with permission from Ref. [61], Copyright 2022, Elsevier) enzymatic electrochemical sensors.

It is well established that metal-based materials (e.g., monometals such as Au, Ag and Cu; bimetals such as AuPt, AuPd and PdRh) have been widely used to construct enzymatic electrochemical sensors due to their excellent electron transfer capability, good catalytic activity and stability, outstanding biocompatibility, high sensitivity and repeatability [47]. Based on the specific interaction between Au and the amino group of AChE, Dhull et al. [48] fixed AChE on SWCNTs using nafion as a crosslinker, followed by coating the composites on Au wire (AW) electrode-decorated by Au NPs and MWCNTs to constructing enzymatic electrochemical sensors (AChE-SWCNTs-Au NPs-MWCNTs-AW) for detecting multiple OPs. A linear relationship between the response current of sensors and the concentration of OPs was observed. The concentration range was 1 nM–46 µM for methyl parathion, 1 nM–52 µM for monocrotophos, 1 nM–52 µM for chlorpyrifos and 20 nM–130 µM for endosulfan. Their corresponding LOD were 1.9 nM, 2.3 nM, 2.2 nM and 2.5 nM, respectively. Using MWCNTs as conductive additives, Citterio et al. [49] modified SPE with Chi and Au NPs composites and immobilized AChE to fabricate enzymatic electrochemical sensors (AChE-Au NPs-MWNTs-SPE) for detecting paraoxon. Under optimized conditions, amperometry analysis revealed two linear relationships between the response current and the concentration of paraoxon. One concentration range was 0.01–10 μg L^−1^, and the other was 10–100 μg L^−1^. The LOD of the sensors was 0.03 μg L^−1^. The high detection performance of sensors was attributed to the synergistic effect between the high conductivity of Au NPs and the high specific surface area of MWCNTs. Additionally, Hou et al. [50] prepared enzymatic electrochemical sensors based on the composites of plant esterase, Chi, Gra and Au NPs (Au NPs-PE-Chi-Gra) for detecting methyl parathion and malathion, as shown in Figure 5a. Under optimal reaction conditions, DPV analysis showed the linear concentration range was 0–200 ppb for methyl parathion (LOD = 0.19 nM) and 0–500 ppb for malathion (LOD = 1.51 nM), respectively. To further improve the specific surface area of Au, Guo et al. [51] designed Au nanocages to load the composites of GO and Chi for SPE modification (Au NCs-GO-Chi), followed by the fixation of AChE to construct enzymatic electrochemical sensors, as shown in Figure 5b. Under optimized conditions, the sensors exhibited high electrochemical performance for detecting chlorpyrifos with a linear concentration range of 0.01–500 μg L^−1^ and an LOD of 3 ng L^−1^. The outstanding detection performance of sensors was related to the synergistic effect between the high specific surface area, good dispersibility and remarkable adhesion of GO and the high specific surface area and excellent electron conductivity of Au nanocages. Furthermore, Cui et al. [52] incorporated the Au NPs and mesoporous SiO_2_ core-shell composites into the matrix of Chi and TiO_2_ hydrogel (Au NPs-SiO_2_-TiO_2_) to modify GCE, followed by the fixation of AChE to construct enzymatic electrochemical sensors, as shown in Figure 5c. Under optimized electrochemical conditions, the sensors displayed high sensitivity for detecting dichlorvos and fenthion. The linear concentration range was 0.018–13.6 μM for both OPs, but the LOD was 5.3 nM for dichlorvos and 1.3 nM for fenthion, respectively.

The hydrolysis of indoxyl acetate catalyzed by AChE produces hydroxyindole, which can promote the electrochemical reduction of Ag^+^ to metallic Ag, generating a detectable response current. Based on this principle, Li et al. [53] designed enzymatic electrochemical sensors for detecting chlorpyrifos by correlating AChE catalytic activity with chlorpyrifos concentration in the electrolyte solution, using the response current from Ag electrodeposition on GE surfaces as a detection signal. Under optimal detection conditions, linear sweep voltammetry (LSV) analysis showed that the peak response current of sensors was positively related with the concentration of chlorpyrifos, in the concentration range of 10 pM–10 nM with an LOD of 4.0 pM. The recovery for chlorpyrifos was 92–104% in lake water. In addition, using the mixtures of Ag nanofibers (Ag NFs) and Gra as conductive additives, Zhang et al. [54] immobilized AChE on GCE modified with TiO_2_ hydrogel and Chi composites to construct enzymatic electrochemical sensors (AChE-TiO_2_-Chi-Ag NFs-Gra-Chi-GCE), as shown in Figure 5d. Under optimal reaction conditions, DPV analysis showed a linear relationship between the response current peak and the concentration of dichlorvos, in the concentration range of 0.036–22.63 mM with an LOD of 7.4 nM. Furthermore, based on the interaction between AChE and amino group-modified CNTs, Gao et al. [55] used Ag NP-decorated nitrogen-fluorine co-doped MoS_2_ (Ag NPs-N-F-MoS_2_) as carriers to immobilize the composites of AChE and CNTs, preparing enzymatic electrochemical sensors. Under optimal detection conditions, DPV analysis confirmed that the sensors exhibited high sensitivity, good stability and excellent reproducibility and selectivity. In the concentration range of 10–10^−6^ mg mL^−1^ for monocrotophos, the peak response current of sensors decreased linearly with the increase in its concentration. However, two linear relationships were observed for chlorpyrifos. One concentration range was 5 × 10^−8^–10^−7^ mg·mL^−1^, and the other was 10^−7^–10^−4^ mg mL^−1^. The LOD was 0.05 pg·mL^−1^ for monocrotophos and 1 pg·mL^−1^ for chlorpyrifos, respectively. Additionally, Gao et al. [56] used three-dimensional Ag tree-like nanostructure (3D Ag TN) as AChE carriers to prepare enzymatic electrochemical sensors for detecting omethoate, as shown in Figure 5e. Under optimal reaction conditions, DPV analysis showed that the peak response current of sensors was inversely proportional to the concentration of omethoate. The linear concentration range was 10^−13^–10^−7^ M. The LOD of sensors was 1.26 × 10^−14^ M. The high detection performance of sensors was attributed to the high conductivity, ultrahigh hydrophilic and biocompatibility of tree-like Ag structures.

As shown in Figure 5f, Kulchat et al. [57] coated the composites of Cu nanowires (Cu NWs), rGO and AChE on the SPE surface to construct enzymatic electrochemical sensors (AChE-Cu NWs-rGO-SPE). Under optimized electrochemical conditions, CV analysis showed the response anodic peak current of sensors decreased with the increase in the concentration of chlorpyrifos. The linear concentration range was 10 µg L^−1^–200 µg L^−1^. The LOD and LQD were 3.1 µg L^−1^ and 12.5 µg L^−1^, respectively. The sensors displayed strong anti-interference capacity against metal ions and satisfactory recovery rates for chlorpyrifos in drinking water and orange juice samples. The excellent electrochemical performance of sensors was ascribed to the high conductivity of Cu NWs and rGO, which facilitated the amplification of the detection signal.

In addition, bimetallic materials have been used to prepare high-performance enzymatic electrochemical sensors for detecting OPs, owing to their synergistic effects. For instance, Huang et al. [58] immobilized the mixtures of AChE and deacetylated Chi on GCE modified with self-interconnected AuPd bimetallic nanowires (AuPd NWs), fabricating enzymatic electrochemical sensors for detecting malathion. Under optimal detection conditions, DPV analysis showed the sensors displayed high sensitivity, good reproducibility, remarkable selectivity and good stability. The peak response current of sensors decreased with the increase in the concentration of malathion, in the concentration range of 0.1 PM to 100 nM. The LOD was 0.037 pM. The high performance of sensors was related to the unique structure of bimetallic nanowires, which facilitated the formation of multiple electron transfer paths and increased the electrochemical active area. Ping et al. [59] coated AuPd bimetallic nanoparticles in MXene (AuPd NPs-MXene)-modified SPE and used glutaraldehyde as crosslinker for AChE immobilization to construct enzymatic electrochemical sensors for the detection of paraoxon, as shown in Figure 5g. Under optimal conditions, amperometry analysis showed that the inhibition rate of the sensors was positively correlation with the concentration of paraoxon. The linear concentration range was 0.1–1000 μg L^−1^. The LOD was 1.75 ng L^−1^. The recovery for paraoxon was 87.93–111.02% in pear and cucumber samples. Benefiting from the ultralow density, abundant porous structure and ultrahigh specific surface area of hydrogels, Wu et al. [60] prepared the composites of polydopamine-coated AuPt bimetallic hydrogels (PD-AuPt) with one-step wet chemical methods to modify GCE, followed by the fixation of AChE to build enzymatic electrochemical sensors, as shown in Figure 5h. Under optimized electrochemical conditions, DPV analysis showed a linear relationship between the response current of sensors and the concentration of paraoxon. The linear concentration range was 0.5–1000 ng L^−1^, and the LOD was 0.185 ng L^−1^. Furthermore, Gao et al. [61] utilized one-dimensional PdRh bimetallic nanotubes (1D PdRh NTs, average diameter of 12 nm and pore size of 8–50 nm) to modify GCE, followed by Chi-mediated AChE immobilization to build enzymatic electrochemical sensors for detection of carbaryl, as shown in Figure 5i. Chronoamperometry analysis showed that the sensitivity of the sensors was 0.279 μA nM. Differential pulse voltammetry analysis displayed a linear relationship between the peak response current of sensors and the concentration of carbaryl from 9.44 × 10^−8^–0.944 mg L^−1^, and the calculated LOD was 9.44 × 10^−8^ mg L^−1^. The sensors exhibited high selectivity for inorganic salt ions. The recovery was 94.01–102.80% for carbaryl in tap and lake water samples. The excellent electrochemical performance of sensors was attributed to the large specific surface area, abundant catalytic activity sites and rapid electron transfer properties of one-dimensional bimetallic nanotubes.

In conclusion, owing to the specific interactions between metal-based materials and bioactive enzymes, metal-based materials have been widely used as enzyme carriers to fabricate enzymatic electrochemical sensors, which exhibit high sensitivity, good selectivity, excellent stability and considerable reproducibility for detecting OPs. However, most reported metal-based materials are precious metals. Therefore, the development of high-performance non-precious metal-based materials is crucial for constructing low-cost enzymatic electrochemical sensors for OP detection. Additionally, rational nanostructure design of metal-based materials is necessary to improve their mass transfer performance and enzyme immobilization capacity, thereby enhancing the electrochemical detection of metal-based enzymatic electrochemical sensors.

### 4.4. Metal Compound-Based Materials

It has been reported that the abundant redox active sites of metal compound-based materials can accelerate electron transfer between bioactive enzymes and working electrodes, thus reducing response time and improving sensor sensitivity [62]. In addition, surface functional groups of metal compound-based materials facilitate bioactive enzyme immobilization through covalent bonding [63]. For these reasons, metal compound-based materials have been applied to build enzymatic electrochemical sensors for detecting OPs. For instance, Gao et al. [64] modified GCE with Au NP-decorated three-dimensional flower-like α-Fe_2_O_3_ and CNT composites (Au-NPs-3D Fe_2_O_3_-CNTs), followed by the fixation of AChE to construct enzymatic electrochemical sensors. Under optimized detection conditions, DPV analysis showed that the sensors displayed excellent sensitivity, high selectivity, considerable stability and remarkable reproducibility for detecting omethoate. A linear relationship between the peak response current of sensors and omethoate concentration was observed in the concentration rang of 10^−13^–10^−7^ M with an LOD of 1.9 × 10^−15^ M. The recoveries were 96.04–100.99% for omethoate in tomato samples. The sensors also displayed ultrahigh sensitivity for detection of other OPs, with an LOD of 1.02 × 10^−15^ M for malathion, 1.34 × 10^−14^ M for fenitrothion and 10^−13^ M for chlorpyrifos, respectively. The outstanding electrochemical performance was attributed to the synergistic effect between the large specific surface area and good biocompatibility of α-Fe_2_O_3_ and the high conductivity of Au NPs and CNTs. In addition, Niu et al. [65] immobilized AChE on GCE modified with magnetic Fe_3_O_4_ and mesoporous carbon sphere core-shell composites (Fe_3_O_4_-MCS) to build enzymatic electrochemical sensors. Under optimized reaction conditions, two linear relationships were observed between the peak response current and the concentration of malathion. One concentration range was 0.01–50 ppb, the other was 50–600 ppb. The calculated LOD was 0.0182 ppb.

**Figure 6:**
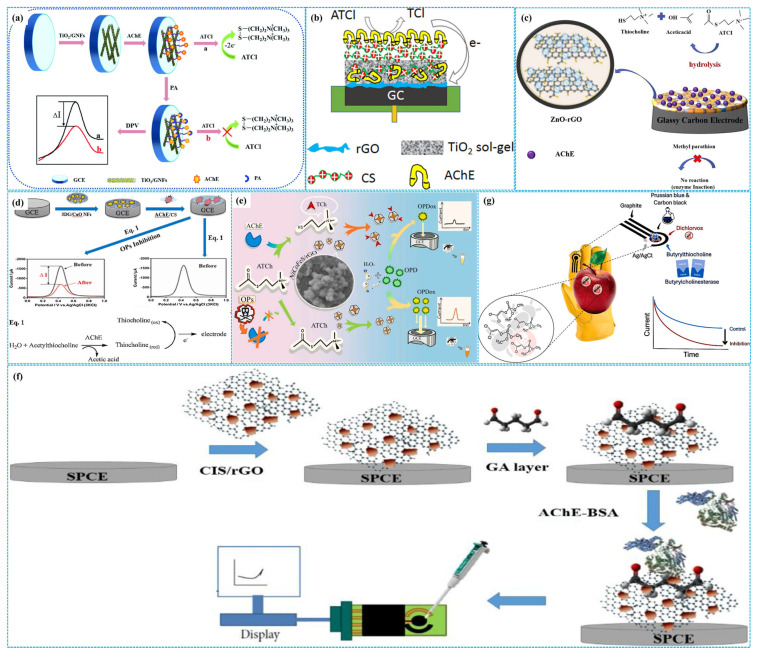
The preparation of TiO_2_−g−GNF−based ((**a**), reprinted with permission from Ref. [68], Copyright 2022, Royal Society of Chemistry), TiO_2_ H−Chi−based ((**b**), reprinted with permission from Ref. [70], Copyright 2018, Elsevier), ZnO−rGO−based ((**c**), reprinted with permission from Ref. [71], Copyright 2024, Elsevier), 3D Gra−CuO NP−based ((**d**), reprinted with permission from Ref. [73], Copyright 2019, Elsevier), NiCoFeS−based ((**e**), reprinted with permission from Ref. [75], Copyright 2025, Elsevier), CuInS_2_−GO−based ((**f**), reprinted with permission from Ref. [76], Copyright 2021, Elsevier) and PB−BC−based ((**g**), reprinted with permission from Ref. [79], Copyright 2025, Elsevier) enzymatic electrochemical sensors.

It is well known that Ti atoms of TiO_2_ can bind to the carboxyl/amino groups of AChE [66] and react with the phosphate groups of OPs [67]. For this reason, Guo et al. [68] utilized graphitized CNF-supported TiO_2_ nanoparticles (TiO_2_-g-CNFs) to modify GCE, followed by AChE fixation to construct enzymatic electrochemical sensors (Figure 6a). Under optimal detection conditions, the response current of sensors was positively correlated with the concentration of paraoxon. The linear paraoxon concentration range was 10^−13^–10^−8^ M, and the LOD was 3.3 fM. The sensors exhibited good selectivity, remarkable reproducibility, high stability and satisfactory recovery rates. The outstanding electrochemical performance was related to the high conductivity derived from the graphitized structure of carbon nanofibers. In addition, Yang et al. [69] incorporated TiO_2_ nanoparticles into Chi matrices (TiO_2_ NPs-Chi) to decorate GCE, followed by coating the mixtures of Chi and AChE on modified electrodes, building patterned enzymatic electrochemical sensors. Under optimized conditions, DPV analysis showed that the peak response current decreased with the increase in the concentration of dichlorvos in the concentration range of 1.13 nM–22.6 μM with an LOD of 0.23 nM. Furthermore, based on electrodeposited composites of Chi and TiO_2_ hydrogels (TiO_2_ H-Chi), Cui et al. [70] immobilized AChE on modified GCE to prepare enzymatic electrochemical sensors, as shown in Figure 6b. Under optimal reaction conditions, the sensors exhibited high sensitivity for detecting dichlorvos. There were two linear relationships between the peak response current and the concentration of dichlorvos. One concentration range was 0.036–0.453 μM, and the other was 0.453–22.6 μM. The calculated LOD of sensors was 29 nM.

Based on the synergistic effect between p- and n-type semiconductors, Niu et al. [71] fixed AChE on GCE decorated with p-n heterojunctions of n-type ZnO nanoparticles and p-type rGO (ZnO-rGO) to build enzymatic electrochemical sensors for the detection of methyl parathion, as shown in Figure 6c. Under optimized detection conditions, DPV analysis indicated that the peak response current decreased with the increase in the concentration of methyl parathion. The linear concentration range was 10^−5^–1 μg mL^−1^ with a sensitivity of 0.004638 μA (ng mL^−1^)^−1^ and an LOD of 0.11871 ng mL^−1^. The recovery for methyl parathion was 90.92–108.4% in apple and cucumber samples. The high detection performance was correlated with the formation of p-n heterojunction, which improved electrical conductivity and reduced the redox reaction potential. In addition, Liu et al. [72] immobilized AChE on GCE modified with core-shell hollow ZnO-CoO and N-PC composites (ZnO-CoO-N-PC). Under optimal electrochemical conditions, the sensors displayed high sensitivity for detecting chlorpyrifos and parathion-methyl. The concentration range was 7.6 × 10^−15^–7.6 × 10^−6^ M for parathion-methyl (LOD = 7.6 × 10^−15^ M) and 2.74 × 10^−13^–2.74 × 10^−6^ M for chlorpyrifos (LOD = 2.74 × 10^−13^ M), respectively. Huo et al. [73] prepared enzymatic electrochemical sensors by immobilizing AChE on GCE modified with the composites of three-dimensional Gra and flower-like CuO nanoparticles (3D Gra-CuO NPs), as shown in Figure 6d. Under optimized electrochemical conditions, SWV analysis showed that the peak response current decreased with increasing the concentration of malathion. A linear relationship was observed in the concentration of 1 ppt–15.555 ppb with an LOD of 0.31 ppt. The sensors displayed good stability, excellent reproducibility and considerable selectivity.

Thiocholines, the hydrolysis products of acetylthiocholine, can accelerate the decomposition of MnO_2_. Based on this mechanism, Tang et al. [74] used thionine as a multiple electron mediator to fabricate split-type homogeneous enzymatic electrochemical sensors for detecting dichlorvos. In these sensors, OP-induced inhibition of bioactive enzyme catalytic activity reduces thionine release, leading to a decrease in the response current change. Under optimal detection conditions, differential pulse voltammetry analysis showed that the peak response current decreased with the increase in the concentration of dichlorvos. The concentration range was 10^−6^–10^−10^ M, and the LOD was 3 × 10^−10^ M. In addition, it is well known that the electrochemical hydrolysis of H_2_O_2_ generates a response current, and the oxidation of o-phenylenediamine produces a color change. Since the specific binding between NiCoFeS nano-enzymes and thiocholines can inhibit their catalytic activity for H_2_O_2_ electrochemical hydrolysis, Zou et al. [75] designed dual-signal (colorimetric and electrochemical) homogeneous enzymatic sensors for detection of trichlorfon based on the controllable synthesis of NiCoFeS nano-enzymes, as shown in Figure 6e. Under optimal conditions, differential pulse voltammetry analysis revealed a linear relationship between the response current change and the concentration of trichlorfon, in the concentration range of 12.5 fg mL^−1^–1.25 ng mL^−1^ with an LOD of 9.74fg mL^−1^. Colorimetric analysis showed a linear correlation between the absorbance and the concentration of trichlorfon, in the concentration range of 0.25 pg mL^−1^–125 pg mL^−1^ with an LOD of 0.12 pg mL^−1^ under the same detection conditions.

In addition, combining the multiple metallic redox sites, abundant catalytic active centers and high specific surface area of CuInS_2_ with the high conductivity of GO, Hasin et al. [76] used glutaraldehyde to fix the composites of CuInS_2_ and GO (CuInS_2_-GO) on SPE, followed by coating the mixtures of AChE and BAS on the modified electrode to build enzymatic electrochemical sensors for detecting chlorpyrifos (Figure 6f). Under optimized conditions, LSV analysis showed that two linear relationships between the response current and the concentration of chlorpyrifos were observed. One linear concentration range was 0.5–230 ng mL^−1^, and the other was 240–470 ng mL^−1^. The LOD was 0.023 ng mL^−1^. The sensors displayed good reproducibility, excellent selectivity and high recovery for chlorpyrifos in vegetable samples. Furthermore, bimetallic oxides have also been used to construct enzymatic electrochemical sensors for OP detection. For example, Gao et al. [77] prepared three-dimensional hierarchical flower-like Co-based bimetallic oxide nanosheets (3D CoXO) using a hydrothermal approach, followed by AChE immobilization on modified GCE to fabricate enzymatic electrochemical sensors. Under optimal conditions, DPV analysis showed that the sensors displayed high sensitivity, good selectivity and excellent stability for detecting diazinon and omethoate. The linear concentration range was 3.88 × 10^−6^–3.88 μM for diazinon (LOD = 0.36 pM) and 4.69 × 10^−7^–0.469 μM for omethoate (LOD = 0.033 pM), respectively.

In addition, Prussian blue (PB) has been used as an active material for constructing enzymatic electrochemical sensors. For instance, Teng et al. [78] used nafion as a crosslinker to immobilize AChE on the surface of SPE modified by PB, constructing dual-channel enzymatic electrochemical sensors. Under optimized detection conditions, the dual-channel sensors displayed high sensitivity for isocarbophos, chlorpyrifos and trichlorfon with an LOD of 10^−7^ g mL^−1^ for all three analytes. Moreover, Cinti et al. [79] modified SPE with PB and CB bioactive mixtures (PB-CB) and immobilized butyrylcholinesterase to construct glove-based portable enzymatic electrochemical sensors for on-site detection dichlorvos on apples and oranges, as shown in Figure 6g. Under optimized parameters, the glove-based sensors achieved an LOD as low as 8 nM. Their sensitivity was significantly higher than other portable sensors, although it remained lower than that of traditional laboratory-based analytical techniques.

In conclusion, various metal compound-based materials have been employed to construct enzymatic electrochemical sensors. Although these sensors display high sensitivity, good stability and considerable reproducibility, their limited intrinsic conduction reduces response speed and sensitivity. Additionally, the uneven distribution of surface functional groups can cause the leakage of immobilized bioactive enzymes. Therefore, to develop high-performance enzymatic electrochemical sensors, it is critical to fabricate hybrid composites to improve the conductivity of metal compound-based materials and to perform surface functional modification to enhance bioactive enzyme immobilization capacity.

### 4.5. MOF-Based Materials

**Figure 7:**
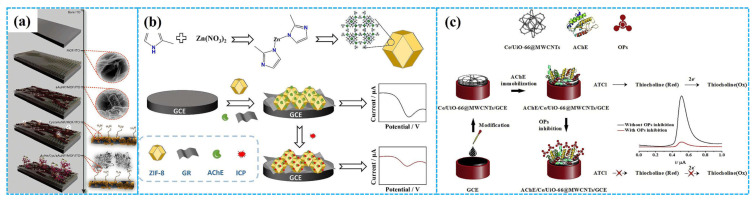
The preparation of MOF−5−based ((**a**), reprinted with permission from Ref. [84], Copyright 2020, Royal Society of Chemistry), Chi−Gra−ZIF−8−based (**b**) and Ce−UiO−66−MWCNT−based ((**c**), reprinted with permission from Ref. [90], Copyright 2019, Elsevier) enzymatic electrochemical sensors.

As we all know, MOFs are constructed by the coordination bonding of metal ions (or clusters) and organic ligands [80]. In October 2025, the Royal Swedish Academy of Sciences awarded the Nobel Prize in Chemistry to Susumu Kitagawa, Richard Robson and Omar Yaghi for their pioneering contributions in the synthesis and functionalization of MOFs, also referred to as porous coordination polymers [81]. In 1989, Robson et al. [82] proposed the concept of porous coordination polymers based on the crystal structure of diamond. In 1995, Yaghi et al. [83] first synthesized MOF-5, a material with a stable three-dimensional open framework structure, using Zn_4_O clusters as secondary building units and terephthalic acid as organic ligands. Due to its unique structural characteristics, MOF-5 has been exploited as the active material of enzymatic electrochemical sensors. For example, Basu et al. [84] adopted an in situ electrochemical approach to deposit anisotropic Au nanorods on the surface of ITOE modified with MOF-5. Cysteamine was used as a crosslinker to immobilize AChE on the modified electrode surface, thereby constructing enzymatic electrochemical sensors for detecting OPs, as shown in Figure 7a. Under optimized reaction conditions, CV analysis revealed a linear relationship between the response anodic current peak of sensors and the concentration of chlorpyrifos, which existed in the concentration range of 30 ng L^−1^–600 ng L^−1^. The sensitivity of sensors was 2.04 µA ng^−1^ L cm^−2^ with an LOD of 3 ng L^−1^. Moreover, the sensors exhibited high sensitivity for detection of five OPs and their mixtures. In addition, the detection performance of these sensors for parathion, methyl parathion, malathion, ethion and chlorpyrifos in abelmoschus esculentus, solanum melongena, capsicum annuum and momordica charantia linn samples was comparable to those of a gas chromatography–electron capture detector.

Furthermore, the Zn^2+^–imidazole coordination moieties in ZIF-8 MOFs endow the material with good biocompatibility, making it suitable for supporting bioactive enzymes [85]. Utilizing Chi as link binders and Gra as conductive additives, Wang et al. [86] coated AChE-Chi-Gra-ZIF-8 composites on GCE to assemble enzymatic electrochemical sensors for detection of isocarbophos, as shown in Figure 7b. Under optimized experimental conditions, differential pulse voltammetry analysis demonstrated that the inhibition ratio of the enzymatic sensors increased with the increase in the concentration of isocarbophos, with a linear concentration range of 1.73–345.7 nM and an LOD of 0.62 nM. Additionally, Zhang et al. [87] employed carboxymethylation materials as link binders to immobilize AChE on amino-functionalized Zn-based MOFs (Zn-MOFs), constructing enzymatic electrochemical sensors for glyphosate detection. Under optimized reaction conditions, DPV analysis indicated that the peak response current decreased with the increase in the concentration of glyphosate, displaying a linear range of 10^−15^–10^−9^ M, LOD of 1.24 × 10^−13^ M and LOQ of 4.13 × 10^−13^ M, respectively. The integration of these sensors with near-field communication chips enabled on-site detection of glyphosate. The outstanding detection performance of the enzymatic sensors is related to the charge redistribution within Zn-based MOFs, which facilitates charge separation between these sensors and glyphosate [88]. This phenomenon is induced by the positively charged amine groups and the abundant hydrogen bond acceptor/donor in the ligand of 5-(5-Amino-3-carboxypyridin-2-yl) isophthalic acid ligands.

In addition, the strong interaction between the Zr ion centers of Zr-based MOFs and the P=O or P=S groups of OPs enable these MOFs to serve as recognition elements of non-enzymatic electrochemical sensors for detecting OPs [89]. Simultaneously, the large specific surface area of Zr-based MOFs makes them ideal carriers for bioactive enzymes in enzymatic electrochemical sensors. To this end, Bagheri et al. [90] anchored AChE onto GCE modified with Ce-UiO-66-MWCNT composites to prepare enzymatic electrochemical sensors for detecting paraoxon, as depicted in Figure 7c. Under optimal detection conditions, DPV analysis showed a linear relationship between the inhibition rate of the enzymatic sensors and the paraoxon concentration, in the concentration range of 0.01–150 nM with an LOD of 0.004 nM. The enzymatic sensors had acceptable stability, good selectivity, remarkable reproducibility and satisfactory recovery rates for paraoxon in cabbage and spinach samples. Their outstanding electrochemical performance for detection of paraoxon was ascribed to the synergistic effect of multiple factors: the multiple valence states and good oxygen affinity of Ce ions [91], the high conductivity of MWCNTs and the high chemical and thermal stability of UiO-66.

In brief, owing to their ultrahigh specific surface area and abundant catalytic active sites, MOFs as active materials for enzymatic electrochemical sensors exhibit high sensitivity and good stability. To further enhance the detection performance of MOF-based enzymatic electrochemical sensors, it is imperative to introduce conductive materials such as PC, Gra and conductive polymers to form hybrid composites, which can accelerate the electron transfer between bioactive enzymes and the working electrode. Additionally, optimizing the pore structure of MOF-based materials can promote the diffusion of substrates and expand the linear detection range of electrochemical sensors.

### 4.6. COF-Based Materials

**Figure 8:**
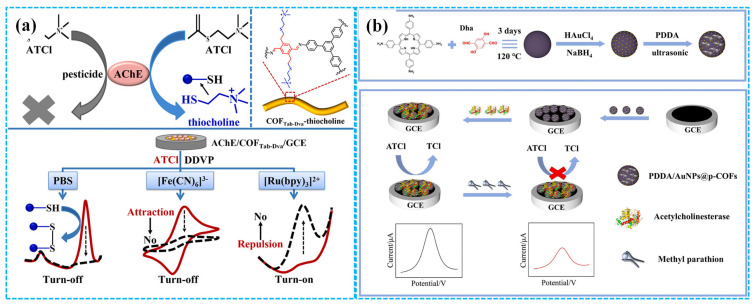
The detection mechanism of the “turn off” and “turn on” strategy ((**a**), reprinted with permission from Ref. [94], Copyright 2022, Elsevier); the preparation of the PDDA-Au NPs-COF-based enzymatic electrochemical sensor ((**b**), reprinted with permission from Ref. [96], Copyright 2024, Elsevier).

Due to their large specific surface area, high porosity, well-defined crystalline structure, considerable chemical and electrochemical stability and remarkable biocompatibility, COFs exhibit great potential for immobilizing bioactive enzymes [92]. However, enzymatic electrochemical sensors using TCh and [Fe(CN)_6_]^3−^ as signal generators (that is “turn off” mechanism) suffer from inadequate selectivity and repeatability [93]. To address these problems, Ma et al. [94] developed “turn on” low potential enzymatic electrochemical sensors by immobilizing AChE on COF NF-modified GCEs, with [Ru(bpy)_3_]^2+^ as a signal generator (Figure 8a). Compared to sensors based on TCh and [Fe(CN)_6_]^3−^, these sensors showed nearly identical catalytic activity and stability for dichlorvos detection while demonstrating significantly enhanced selectivity and repeatability. Alternatively, Wang et al. [95] synthesized core-shell COFs-MWCNT composites using 1,3,5-Tris(4-aminophenyl) benzene and 2,5-divinylterephthalaldehyde as organic building blocks. These composites were used for modifying GCE, followed by AChE immobilization for malathion detection. Electrochemical analysis revealed a linear relationship between the oxidation current of acetylthiocholine chloride and its concentration under optimized conditions. The linear concentration range was 1 nM–10 μM, and the LOD was 0.5 nM. The recovery was 96.0–101.6% for tap water and 98.0–105.0% for spinach samples, respectively.

Taking advantage of the synergistic effect of the conductivity of Au NPs, the conjugated π-electron macrocycles of porphyrin-based COFs and the electrostatic interaction between the positively charged poly diallyldimethylammonium chloride (PDDA) and the negatively charged AChE, Zhang et al. [96] immobilized AChE on PDDA-Au NPs-COF composite-modified GCE to fabricate enzymatic electrochemical sensors for methyl parathion detection (Figure 8b). DPV analysis revealed that these sensors exhibited remarkable sensing performances for methyl parathion detection, including outstanding electrochemical activity, good stability, excellent repeatability and high selectivity. A linear relationship between the inhibition efficiency of enzymatic electrochemical sensors and the concentration of methyl parathion was observed in the concentration range of 1.9 × 10^−9^–3.8 × 10^−5^ M with an LOD of 2.3 × 10^−10^ M. The recovery for methyl parathion was 95.7–107.9% in tomato and strawberry samples. The introduction of Au NPs and PDDA reduced the electron transfer resistance of modified electrodes, thus improving the detection ability of enzymatic electrochemical sensors. Separately, Song et al. [97] prepared enzymatic electrochemical sensors based on the composites of biomass PC and COFs (BPC-COFs) for trichlorfon detection. Under optimized conditions, DPV analysis showed the response current peak of the sensors decreased with the increase in the concentration of trichlorfon, in the concentration range of 0.2–19 ng mL^−1^. The calculated LOD was 0.067 ng mL^−1^.

In conclusion, COF-based enzymatic electrochemical sensors have displayed high sensitivity for OP detection. However, their performance is constrained by four key limitations: (1) poor intrinsic conductivity of COF-based materials hinders electron transfer between the bioactive enzyme and the working electrode, resulting in the weak response signals; (2) bioactive enzyme-induced channel blockage of COF-based materials causes the inhomogeneous distribution of bioactive enzymes and hinders the diffusion of substrates; (3) the complex preparation process of COF-based materials requires toxic organic solvent; and (4) the residual organic solvent and monomers in COF-based materials may inactivate immobilized bioactive enzymes. Therefore, addressing these challenges is significant for developing high-performance COF-based enzymatic electrochemical sensors.

Overall, based on the aforementioned literature, the GCE is the most commonly preferred working electrode for fabricating lab-scale enzymatic electrochemical sensors, attributed to its high chemical inertness, low cost and facile modifiability. However, GCE is difficult to integrate with alternative supporting substrates such as paper- or glove-based substrates, which limits its applicability for achieving on-site detection of OPs. From this point of view, as compared with other working electrodes, SPE fabricated via screen-printing technology represents the ideal candidate for building practical enzymatic electrochemical sensors and enabling on-site OP detection. The primary challenge hindering the practical application of SPE-based enzymatic electrochemical sensors lies in reducing contact resistance and enhancing electrical conductivity.

In addition, Table 2 comprehensively summarizes the electrochemical active materials, analytical techniques and electrochemical detection performances of reported enzymatic electrochemical sensors for OP analysis. As shown in Table 2, a variety of analytical techniques have been employed to evaluate the detection performance of enzymatic electrochemical sensors, including DPV, CV, LSV, SWV, electrochemical impedance spectroscopy, amperometry and chronoamperometry. Among them, DPV is the most widely used technique for OP detection. This method combines linear scanning voltammetry with pulse technology, endowing it with numerous advantages: (1) a lowered LOD, making it suitable for trace-level analysis; (2) a well-resolved redox potential window, enabling simultaneous detection of multiple analytes; (3) excellent anti-interference capacity against inert electrolytes and trace impurities in test solutions, accompanied by a stable baseline; (4) a favorable balance between detection sensitivity and efficiency, rendering it applicable for batch sample analysis; and (5) the ability to detect reversible/semi-reversible electroactive species. Despite these merits, DPV also has several inherent drawbacks: low sensitivity toward irreversible electroactive species and inapplicability for rapid dynamic detection. It is therefore necessary to select an appropriate analytical technique based on the practical application scenarios of enzymatic electrochemical sensors.

As is well established, the key parameters for evaluating the detection performance of enzymatic electrochemical sensors toward OP include the linear concentration range, LOD, LOQ, sensitivity, selectivity, storage stability, repeatability, reproducibility, reusability and recovery rate in real samples, all of which are also systematically summarized in Table 2. Specifically, the linear concentration range and LOD represent the fundamental metrics for the performance evaluation of enzymatic electrochemical sensors. Through the unremitting efforts of researchers, enzymatic electrochemical sensors have been demonstrated to exhibit a broad linear concentration range down to the nanomolar or micromolar level, accompanied by an ultralow LOD reaching the picomolar scale. These sensors have also shown excellent repeatability and reproducibility, with the RSD consistently below 10%, suggesting their preparation process is well optimized and suitable for batch production. In addition, enzymatic electrochemical sensors have displayed robust anti-interference capability against inorganic ions, low-molecular-weight organic acids and sugars. However, their selectivity toward OPs remains relatively poor, which is attributable to the intrinsic broad-spectrum catalytic activity of the enzymes employed for OP detection. Therefore, the development of selective sensors is of significance for the specific detection of OPs. Furthermore, storage stability represents another crucial performance parameter of enzymatic electrochemical sensors. As shown in Table 2, the vast majority of storage stability assessments have been conducted at 4 °C over a 30-day period. Even under such optimized storage conditions, the storage stability of these sensors was still unsatisfactory, as some sensors retained merely 40–50% of their initial activity after storage. Although reactivation treatments could restore the electrochemical activity of these sensors, the recovery efficiency was only approximately 90%. Accordingly, it is imperative to develop effective strategies to enhance the storage stability of enzymatic electrochemical sensors. Finally, the recovery rates of these sensors in real samples exhibit a wide distribution, so it is necessary to suppress this distribution for obtaining detection performance comparable to that of chromatographic techniques.

## 5. Conclusions and Outlooks

This review comprehensively discusses the recent advances in enzymatic electrochemical sensors for the detection of OPs. First, the fundamental information of commonly used OPs is summarized. Based on their toxicity, stability and solubility, acephate, glyphosate and trichlorfon are identified as ideal OP models for evaluating the detection performance of enzymatic electrochemical sensors. The structure of such sensors, including electrodes, electrochemical active layers and bioactive enzyme layers, is systematically reviewed. Particularly, active materials (such as carbon-based, polymer-based, metal-based, metal compound-based, MOF-based and COF-based materials), which serve as the core components of enzymatic electrochemical sensors, are emphatically discussed. It is found that enzymatic electrochemical sensors based on these active materials exhibit ultrahigh sensitivity (with a broad linear concentration range down to the nanomolar or micromolar level and an LOD reaching the picomolar scale), acceptable stability (retaining approximately 85–95% of their initial activity after 30 days of storage at 4 °C), excellent repeatability and reproducibility (with the RSD consistently below 10%) and satisfactory recovery rates for OP detection (ranging roughly from 90% to 115%). In addition, due to their inherently high specific area, well-defined crystalline and porous structures, MOF- and COF-based materials are considered as one of the most important active materials for enzymatic electrochemical sensors. However, research on MOF- and COF-based enzymatic electrochemical sensors is still in its infancy. To further promote the practical application of such sensors, future efforts should focus on developing green and eco-friendly synthesis methods to prepare MOF- and COF-based materials and improving their intrinsic conductivity. Furthermore, since the detection mechanism relies on the inhibition of AChE activity by the phosphate groups of OPs, this type of biosensor demonstrates broad-spectrum applicability to all OPs. The specificity for detecting different OPs can be enhanced by employing alternative biomolecules as signal recognition elements. On the other hand, from the perspective of storage stability, the development of non-enzymatic electrochemical sensors is of great significance for OP detection.

## Figures and Tables

**Table 1:** The chemical name, molecular formula and physicochemical properties of commonly used OPs.

OPs	Chemical Name	Molecular Formula	Property
Acephate	N-[methoxy(methylsulfanyl)phosphoryl]acetamide	C_4_H_10_NO_3_PS	White crystalline powder, low toxicity and systematic insecticide, slow hydrolysis in alkaline condition and extremely stable in acidic/neutral condition, photostable and thermotolerant, low volatility, extremely soluble in water, soluble in organic solvent, slight soluble in non-polar solvent
Glyphosate	2-(phosphonomethylamino)acetic acid	C_3_H_8_NO_5_P	White crystalline powder, low toxicity and systematic herbicide, slow hydrolysis in alkaline condition and stable in acidic/neutral condition, photostable, low volatility, extremely soluble in water, insoluble in organic and non-polar solvent
Malathion	diethyl 2-dimethoxyphosphinothioylsulfanylbutanedioate	C_10_H_19_O_6_PS_2_	Colorless to pale yellow oily liquid, low toxicity, rapid hydrolysis in alkaline condition and stable in acidulous/neutral condition, photosensitive, low volatility, soluble in organic solvent, slight soluble in water and mineral oils
Phoxim	N-diethoxyphosphinothioyloxybenzenecarboximidoyl cyanide	C_12_H_15_N_2_O_3_PS	Light yellow oily liquid, low toxicity, rapid hydrolysis in alkaline condition and stable in acidulous/neutral condition, high photosensitive, low volatility, soluble in organic solvent, slight soluble in aliphatic hydrocarbons and insoluble in water
Pirimiphos methyl	4-dimethoxyphosphinothioyloxy-N,N-diethyl-6-methylpyrimidin-2-amine	C_11_H_20_N_3_O_3_PS	Colorless crystalline solid, low to middle toxicity, slow hydrolysis in alkaline condition and stable in acidic/neutral condition, photostable and thermotolerant, low volatility, soluble in aliphatic hydrocarbons and organic solvent, insoluble in water
Trichlorfon	2,2,2-trichloro-1-dimethoxyphosphorylethanol	C_4_H_8_Cl_3_O_4_P	White crystalline powder, low to middle toxicity, rapid isomerization in alkaline condition and stable in acidic condition, photostable and thermotolerant, low volatility, extremely soluble in water, soluble in organic solvent, slight soluble in nonpolar solvent
Chlorpyrifos	diethoxy-sulfanylidene-[(3,5,6-trichloro-2-pyridinyl)oxy]-lambda5-phosphane	C_9_H_11_C_l3_NO_3_PS	White crystalline solid, middle toxicity and broad-spectrum insecticide, hydrolysis in alkaline condition, photosensitive, low volatility, soluble in organic solvent, insoluble in water
Dimethoate	2-dimethoxyphosphinothioylsulfanyl-N-methylacetamide	C_5_H_12_NO_3_PS_2_	White crystalline powder, middle toxicity and systematic insecticide, rapid hydrolysis in alkaline condition and stable in acidic/neutral condition, photosensitive, low volatility, soluble in organic solvent, slight soluble in aliphatic hydrocarbons and water
Fenitrothion	dimethoxy-(3-methyl-4-nitrophenoxy)-sulfanylidene-lambda5-phosphane e	C_9_H_12_NO_5_PS	Light yellow crystalline solid, middle toxicity, hydrolysis in alkaline condition, photosensitive, low volatility, soluble in organic solvent, slight soluble in aliphatic hydrocarbons, insoluble in water
Profenofos	4-bromo-2-chloro-1-[ethoxy(propylsulfanyl)phosphoryl]oxybenzene	C_11_H_15_BrClO_3_PS	Light yellow liquid, middle toxicity and broad-spectrum insecticide, rapid hydrolysis in alkaline condition and stable in acidulous/neutral condition, photostable and thermotolerant, low volatility, soluble in organic solvent, insoluble in water
Diazinon	diethoxy-(6-methyl-2-propan-2-ylpyrimidin-4-yl)oxy-sulfanylidene-lambda5-phosphane	C_12_H_21_N_2_O_3_PS	Colorless to pale yellow oily liquid, middle toxicity and broad-spectrum insecticide, rapid hydrolysis in alkaline condition and stable in acidulous/neutral condition, photosensitivity, low volatility, soluble in organic solvent, insoluble in water
Dichlorvos	2,2-dichloroethenyl dimethyl phosphate	C_4_H_7_C_l2_O_4_P	Colorless to pale yellow transparent liquid, high toxicity and broad-spectrum insecticide and fumigant, rapid hydrolysis in alkaline condition and stable in acidulous/neutral condition, photosensitive and thermosensitive, high volatility, extremely soluble in organic solvent, soluble in water
Ethion	diethoxyphosphinothioylsulfanylmethylsulfanyl-diethoxy-sulfanylidene-lambda5-phosphane	C_9_H_22_O_4_P_2_S_4_	Colorless to pale yellow oily liquid, high toxicity and broad-spectrum insecticide and acaricide, rapid hydrolysis in alkaline condition and stable in acidulous/neutral condition, photosensitive and thermosensitive, low volatility, soluble in organic solvent, insoluble in water
Isocarbophos	propan-2-yl 2-[amino(methoxy)phosphinothioyl]oxybenzoate	C_11_H_16_NO_4_PS	Colorless and transparent oily liquid, high toxicity and systematic insecticide, rapid hydrolysis in alkaline condition and stable in acidulous condition, photosensitive, low volatility, soluble in organic solvent, insoluble in water
Methamidophos	[amino(methylsulfanyl)phosphoryl]oxymethane	C_2_H_8_NO_2_PS	White needle-like crystal, high toxicity and broad-spectrum insecticide, rapid hydrolysis in alkaline condition and stable in acidulous condition, photosensitive and thermosensitive, low volatility, extremely soluble in water, soluble in organic solvent, slight soluble in non-polar solvent
Methomyl	methyl N-(methylcarbamoyloxy)ethanimidothioate	C_5_H_10_N_2_O_2_S	White crystalline powder, high toxicity and carbamate insecticide, rapid hydrolysis in alkaline condition, photosensitive and thermosensitive, low volatility, extremely soluble in water, soluble in organic solvent, slight soluble in non-polar solvent
Methyl Parathion	dimethoxy-(4-nitrophenoxy)-sulfanylidene-lambda5-phosphane	C_8_H_10_NO_5_PS	Colorless crystal, high toxicity, rapid hydrolysis in alkaline condition, photosensitive, low volatility, extremely soluble in organic solvent, insoluble in water
Monocrotophos	dimethyl [(E)-4-(methylamino)-4-oxobut-2-en-2-yl] phosphate	C_7_H_14_NO_5_P	Colorless crystalline solid, high toxicity and systematic insecticide, rapid hydrolysis in alkaline condition and stable in acidulous condition, photosensitive and thermosensitive, low volatility, extremely soluble in water, soluble in organic solvent
Omethoate	2-dimethoxyphosphorylsulfanyl-N-methylacetamide	C_5_H_12_NO_4_PS	Colorless and transparent oily liquid, high toxicity and systematic insecticide, rapid hydrolysis in alkaline condition and stable in acidulous/neutral condition, photosensitive, low volatility, soluble in water and organic solvent, slight soluble in non-polar solvent
Parathion	diethoxy-(4-nitrophenoxy)-sulfanylidene-lambda5-phosphane	C_10_H_14_NO_5_PS	Colorless needle-like crystal, high toxicity, rapid hydrolysis in alkaline condition, photosensitivity, low volatility, soluble in organic solvent, insoluble in water
Paraoxon	diethyl (4-nitrophenyl) phosphate	C_10_H_14_NO_6_P	Colorless to pale yellow crystalline solid, high toxicity, rapid hydrolysis in alkaline condition and stable in acidulous/neutral condition, photosensitive, volatility, soluble in organic solvent, polar and non-polar solvent, insoluble in water

**Table 2:** The electrochemical active materials, analytical techniques and electrochemical detection performance of enzymatic electrochemical sensors.

Electrochemical Active Materials	Analytical Techniques	Electrochemical Detection Performance	Ref.
Gra-Chi	DPV	**Linear concentration range** for 11 OPs: 1–1500 ng⋅mL^−1^; **LOD**: 0.012–0.23 ng⋅mL^−1^; **Repeatability**: relative standard deviation (RSD) range of 1.35–6.69% (3 times); **Stability**: standard deviation (SD) of 6.7% (10 days, 37.4 °C); **Recovery rate** in apple samples: 87.24–110.2%.	[31]
MWCNTs	CV	**Linear concentration range** for paraoxon: 10–50 nM; **LOD**: 0.1 nM; **Anti-interference ability**: ascorbic acid, uric acid, nitrobenzene, nitrophenol, inorganic ions, fructose and sucrose; **Repeatability**: SD of 0.071 (3 times); **Stability**: 54.52% of initial activity (32 days, 4 °C); **Reactivation**: 90.68% of initial activity (15 min, 1.0 mM pralidoxime iodide solution); **Recovery rate** in potato and tap water samples: 97.28–103.56%.	[29]
SWCNTs	SWV	**Linear concentration range** for methyl parathion: 1 × 10^−10^−5 × 10^−6^ M; **LOD**: 3.75 × 10^−11^ M; **Anti-interference ability**: glucose, uric acid, oxalic acid, ascorbic acid and orthophosphoric acid; **Repeatability**: RSD of 0.89% (3 times); **Stability**: 93.14% of initial activity (33 days, 4 °C); **Reproducibility**: RSD of 4.68% (3 electrodes); **Recovery rate** in strawberry and apple samples: 98.31–102.22%.	[32]
PGO	DPV	**Linear concentration range** for paraoxon: 10–45 ng mL^−1^; **LOD**: 1.58 ng mL^−1^; **Anti-interference ability**: citric acid, oxalic acid, glucose, p-toluenesulfonic acid, toluene, p-nitrophenol and carbofuran; **Repeatability**: RSD of 3.6% (6 times); **Stability**: 97.2% of initial activity (30 days, 4 °C); **Reproducibility**: RSD of 1.1% (6 electrodes); **Reactivation**: 96.74% of initial activity (20 min, 0.1 M PBS solution); **Recovery rate** in lettuce and cabbage samples: 96.9–100.4%.	[33]
ATO-Chi-MC	DPV	**Linear concentration range** for chlorpyrifos and methamidophos: 0.01–10^5^ μg L^−1^; **LOD**: 0.01 μg L^−1^ (chlorpyrifos) and 1 μg L^−1^ (methamidophos); **Stability**: 80% of initial activity (30 days, 4 °C); **Reproducibility**: RSD of 6.3% (5 electrodes); **Recovery rate** in oilseed rape, lettuce and Chinese cabbage samples: 91–107%.	[34]
CB	Amperometry	**Linear concentration range** for OPs: 0.7 × 10^−9^–1.4 × 10^−8^ mol L^−1^ (chlorpyrifos) and 1.1 × 10^−9^–2.3 × 10^−8^ mol L^−1^ (carbofuran); **LOD**: 6 × 10^−10^ mol L^−1^ (chlorpyrifos) and 4 × 10^−10^ mol L^−1^ (carbofuran); Stability: 86.6% of initial activity (14 days, 4 °C); **Reproducibility**: RSD range of 2.3–8.6%; **Recovery rate** in olive oil samples: 50–80%.	[35]
N-PC	Chronoamperometry	**Linear concentration range** for dichlorvos and fenitrothion: 10^−10^–10^−5^ g L^−1^; **LOD**: 1.50 pg L^−1^ (dichlorvos) and 4.42 pg L^−1^ (fenitrothion); **Anti-interference ability**: inorganic ions; **Repeatability**: RSD of 1.07% (6 times); **Stability**: 92.3% of initial activity (30 days, 4 °C); **Reproducibility**: RSD of 5.38% (6 electrodes); **Reactivation**: 93.0% of initial activity (8 min, 5.00 mmol·L^−1^ pralidoxime iodide solution); **Recovery rate** in real samples: 96.1–100% (dichlorvos, lettuce leaves) and 90.8–107% (fenitrothion, shanghaiqing leaves).	[36]
Ag-CuO-PANI	DPV	**Linear concentration range** for paraoxon: 5–100 pM; **LOD**: 11.35 pM; **Sensitivity**: 0.5536 μA (pM)^−1^ cm^−2^; **Anti-interference ability**: inorganic ions, ascorbic acid, dopamine and uric acid; **Stability**: 71.3% of initial activity (20 days, 4 °C); **Reproducibility**: RSD of 1.74% (5 electrodes); **Reactivation**: 93.0% of initial activity (8 min, 5.00 mmol·L^−1^ pralidoxime iodide solution); **Recovery rate** in banana, tomato and soil samples: 92–111%.	[38]
Chi-PANI NFs-CNTs	Electrochemical impedance spectroscopy	**Linear concentration range** for paraoxon-methyl: 1 ppt–100 ppb; **LOD**: 0.304 ppt; **Anti-interference ability**: inorganic ions, amino acids, citric acid and glucose; **Repeatability**: RSD of less than 5% (6 times); **Reproducibility**: RSD of 1.74% (5 electrodes); **Recovery rate** in river water, well water, orange juice, grape juice, milk and beef samples: 90–112%.	[40]
Au NPs-MoS_2_-rGO-PI	DPV	**Linear concentration range** for paraoxon: 0.005–0.150 μg mL^−1^; **LOD**: 0.0014 μg mL^−1^; **Sensitivity**: 4.44 μA (μg mL^−1^)^−1^; **Stability**: 96.0% of initial activity (7 days, 4 °C); **Reproducibility**: RSD of 4.68% (5 electrodes); **Recovery rate** in vegetable leave samples: 99–104%.	[41]
Au NPs-IC-PPY	CV	**Linear concentration range** for methyl parathion: 1.3 × 10^−7^–1.0 × 10^−3^ mol L^−1^; **LOD**: 24 fmol L^−1^; **Sensitivity**: 14 μA cm^−2^ g^−1^ mL; **Stability**: 84% of initial activity (7 days, −15 °C).	[43]
DS-IC-PPY	Chronoamperometry	**Linear concentration range** for carbaryl: 0.05–0.25 ng mL^−1^; **LOD**: 0.033 ng mL^−1^ cm^−2^; **Sensitivity**: −59.5 × 10^3^ A cm^−2^ mL g^−1^; LOQ: 0.11 ng cm^2^ mL^−1^; **Repeatability**: RSD of 1.8% (4 times); **Stability**: 40% of initial activity (30 days, 4 °C); **Reproducibility**: RSD of 3.7% (3 electrodes); **Recovery rate** in tap water samples: 114–120%	[44]
Au NPs-Gra-PEDOT-PSS	DPV	**Linear concentration range** for chlorpyrifos: 0.1–10 nM; **LOD**: 0.07 nM; **Anti-interference ability**: inorganic ions, ascorbic acid, citric acid, uric acid and glucose; **Stability**: 91.94% of initial activity (56 days, 4 °C); **Reproducibility**: RSD of 2.45% (9 electrodes); **Recovery rate** in cabbage samples: ~106%.	[45]
Ag NPs-rGO-FBThF	CV	**Linear concentration range** for OPs: 0.099–9.9 μg L^−1^ (malathion) and 0.0206–2.06 μg L^−1^ (trichlorfon); **LOD**: 0.032 μg L^−1^ (malathion) and 0.001 μg L^−1^ (trichlorfon); **Stability**: 82.7% of initial activity (30 days, 4 °C); **Reproducibility**: RSD of 2.30% (malathion) and 3.44% (trichlorfon) (5 electrodes); **Recovery rate** in tap water samples: 95.2–108%.	[46]
SWCNTs-Au NPs-MWCNTs	Amperometry	**Linear concentration range** for OPs: 1 nM–46 µM (methyl parathion), 1 nM–52 µM (monocrotophos), 1 nM–52 µM (chlorpyrifos) and 20 nM–130 µM (endosulfan); **LOD**: 1.9 nM (methyl parathion), 2.3 nM (monocrotophos), 2.2 nM (chlorpyrifos) and 2.5 nM (endosulfan); **Anti-interference ability**: inorganic ions, glucose, fructose, sucrose and ascorbic acid; **Stability**: 70% of initial activity (60 days).	[48]
Au NPs-MWCNTs	Amperometry	**Linear concentration range** for paraoxon: 0.01–10 μg L^−1^ and 10–100 μg L^−1^; **LOD**: 0.03 μg L^−1^; **Stability**: 83% of initial activity (49 days, 4 °C); **Reproducibility**: RSD of 3.02% (10 electrodes); **Recovery rate** in spinach samples: −104%.	[49]
Au NPs-PE-Chi-Gra	DPV	**Linear concentration range** for OPs: 0–200 ppb (methyl parathion) and 0–500 ppb (malathion); **LOD**: 0.19 nM (methyl parathion) and 1.51 nM (malathion); **Anti-interference ability**: inorganic ions, glucose and citric acid; **Stability**: 73% of initial activity (15 days, 4 °C); **Recovery rate** in carrot and apple samples: 92–107.40% (methyl parathion) and 93–109.50% (malathion).	[50]
Au NCs-GO-Chi	DPV	**Linear concentration range** for chlorpyrifos: 0.01–500 μg L^−1^; **LOD**: 3 ng L^−1^; **Anti-interference ability**: inorganic ions; **Stability**: 81% of initial activity (30 days, 4 °C); **Reproducibility**: RSD of 2.8% (10 electrodes); **Recovery rate** in spinach, oilseed rape and lettuce samples: 96.12–101.68%.	[51]
Au NPs-SiO_2_-TiO_2_	DPV	**Linear concentration range** for dichlorvos and fenthion: 0.018–13.6 μM; **LOD**: 5.3 nM (dichlorvos) and 1.3 nM (fenthion); **Stability**: 98% of initial activity (30 days, −4 °C); **Reproducibility**: RSD of 3.2% (4 electrodes); **Recovery rate** in cabbage juice samples: −106%.	[52]
Electrodeposited Ag	LSV	**Linear concentration range** for chlorpyrifos: 10 pM–10 nM; **LOD**: 1 4.0 pM; **Reproducibility**: RSD of 7.8% (10 electrodes); **Recovery rate** in lake water samples: 92–104%.	[53]
TiO_2_-Chi-Ag NFs-Gra-Chi	DPV	**Linear concentration range** for dichlorvos: 0.036–22.63 mM; **LOD**: 7.4 nM; **Anti-interference ability**: inorganic ions and glucose; **Reproducibility**: RSD of 2.128% (3 electrodes); **Recovery rate** in lettuce juice samples: 92.7–110.9%.	[54]
Ag NPs-N-F-MoS_2_	DPV	**Linear concentration range** for OPs: 10–10^−6^ mg mL^−1^ (monocrotophos), 5 × 10^−8^–10^−7^ mg·mL^−1^ and 10^−7^–10^−4^ mg mL^−1^ (chlorpyrifos); **LOD**: 0.05 pg·mL^−1^ (monocrotophos) and 1 pg·mL^−1^ (chlorpyrifos); **Anti-interference ability**: inorganic ions, ascorbic acid, glucose, citric acid, carbaryl and 2-nitrophenol; **Repeatability**: RSD of 2.4% (6 times); **Stability**: 93.7% of initial activity (30 days, 4 °C); **Recovery rate** in fruit samples: 94.6–104.2%.	[55]
3D Ag TN	DPV	**Linear concentration range** for omethoate: 10^−13^–10^−7^ M; **LOD**: 1.26 × 10^−14^ M; **Anti-interference ability**: inorganic ions and glucose; **Repeatability**: RSD of 2.4% (7 times); **Stability**: 91.3% of initial activity (15 days, 4 °C); **Reproducibility**: RSD of 3.9% (7 electrodes); **Recovery rate** in tap water samples: 97.2–102%	[56]
Cu NWs-rGO	CV	**Linear concentration range** for chlorpyrifos: 10 µg L^−1^–200 µg L^−1^; **LOD**: 3.1 µg L^−1^; LQD: 12.5 µg L^−1^; **Anti-interference ability**: inorganic ions; **Recovery rate** in water and orange juice samples: 96.67–105.65%	[57]
AuPd NWs	DPV	**Linear concentration range** for malathion: 0.1 PM–100 nM; **LOD**: 0.037 pM; **Anti-interference ability**: inorganic ions, carbamide, glucose and citric acid; **Repeatability**: RSD of 1.78% (5 times); **Stability**: 91% of initial activity (30 days, 4 °C); **Reproducibility**: RSD of 3.86% (6 electrodes); **Recovery rate** in tap water samples: 97.8–103%.	[58]
AuPd NPs-MXene	Amperometry	**Linear concentration range** for paraoxon: 0.1–1000 μg L^−1^; **LOD**: 1.75 ng L^−1^; **Anti-interference ability**: inorganic ions, ethyl-paraoxon, ediphenphos, fenitrothion, phenol, ascorbic acid and glucose; **Stability**: 95% of initial activity (7 days, 4 °C); **Recovery rate** in pear and cucumber samples: 87.93–111.02%.	[59]
PD-AuPt	DPV	**Linear concentration range** for paraoxon: 0.5–1000 ng L^−1^; **LOD**: 0.185 ng L^−1^; **Anti-interference ability**: inorganic ions, urea and glucose; **Recovery rate** in tap water and lake water samples: 94.63–103.46%.	[60]
1D PdRh NTs	DPV	**Linear concentration range** for carbaryl: 9.44 × 10^−8^–0.944 mg L^−1^; **LOD**: 9.44 × 10^−8^ mg L^−1^; **Sensitivity**: 0.279 μA nM; **Anti-interference ability**: inorganic ions, glucose, ascorbic acid, citric acid and urea; **Repeatability**: RSD of 1.65% (8 times); **Stability**: 91.1% of initial activity (30 days, 4 °C); **Reproducibility**: RSD of 2.29% (8 electrodes); **Recovery rate** in tap and lake water samples: 94.01−102.80%.	[61]
Au NPs-3D Fe_2_O_3_-CNTs	DPV	**Linear concentration range** for omethoate, malathion, fenitrothion and chlorpyrifos: 10^−13^–10^−7^ M; **LOD**: 1.9 × 10^−15^ M (omethoate), 1.02 × 10^−15^ (malathion), 1.34 × 10^−14^ M (fenitrothion) and 10^−13^ M (chlorpyrifos); **Anti-interference ability**: inorganic ions, glucose, urea and ascorbic acid; **Repeatability**: RSD of 1.69% (5 times); **Stability**: 91.3% of initial activity (30 days, 4 °C); **Reproducibility**: RSD of 2.25% (5 electrodes); **Recovery rate** in real samples: 96.04–100.99%.	[64]
Fe_3_O_4_-MCS	DPV	**Linear concentration range** for malathion: 0.01–50 ppb and 50–600 ppb; **LOD**: 0.0182 ppb; **Anti-interference ability**: inorganic ions; **Repeatability**: RSD of 6.9% (6 times); **Stability**: 79% of initial activity (30 days, 4 °C); **Reproducibility**: RSD of 6.4% (6 electrodes); **Recovery rate** in pear samples: 97.80–104.10%.	[65]
TiO_2_-g-CNFs	DPV	**Linear concentration range** for paraoxon: 10^−13^–10^−8^ M; **LOD**: 3.3 fM; **Anti-interference ability**: inorganic ions and glucose; **Stability**: 96.5% of initial activity (30 days); **Reproducibility**: RSD of 3.7% (6 electrodes); **Recovery rate** in lake water samples: 96–101%.	[68]
TiO_2_ NPs-Chi	DPV	**Linear concentration range** for dichlorvos: 1.13 nM–22.6 μM; **LOD**: 0.23 nM; **Anti-interference ability**: inorganic ions and glucose; **Recovery rate** in cabbage juice samples: 97.4–110%.	[69]
TiO_2_ H-Chi	DPV	**Linear concentration range** for dichlorvos: 0.036–0.453 μM and 0.453–22.6 μM; **LOD**: 29 nM; **Repeatability**: RSD of 1.49% (4 times); **Stability**: 100% of initial activity (30 days); **Recovery rate** in cabbage juice samples: −103%.	[70]
ZnO-rGO	DPV	**Linear concentration range** for methyl parathion: 10^−5^–1 μg mL^−1^; **LOD**: 0.11871 ng mL^−1^; **Sensitivity**: 0.004638 μA (ng mL^−1^)^−1^; **Recovery rate** in apple and cucumber samples: 90.92–108.4%.	[71]
ZnO-CoO-N-PC	DPV	**Linear concentration range** for OPs: 7.6 × 10^−15^–7.6 × 10^−6^ M (parathion-methyl) and 2.74 × 10^−13^–2.74 × 10^−6^ M (chlorpyrifos); **LOD**: 7.6 × 10^−15^ M (parathion-methyl) and 2.74 × 10^−13^ M (chlorpyrifos); **Anti-interference ability**: inorganic ions, glucose, urea and sodium citrate; **Repeatability**: RSD of 2.22% (6 times); **Stability**: 90% of initial activity (15 days, 4 °C); **Reproducibility**: RSD of 3.07% (6 electrodes).	[72]
3D Gra-CuO NPs	SWV	**Linear concentration range** for malathion: 1 ppt–15.555 ppb; **LOD**: 0.31 ppt; **Anti-interference ability**: inorganic ions, glucose, methyl parathion and deltamethrin; **Stability**: 93.70% of initial activity (20 days, 4 °C); **Reproducibility**: RSD of 4.7% (5 electrodes); **Recovery rate** in water samples: 94.33–106.83%.	[73]
MnO_2_-based homogeneous sensor	DPV	**Linear concentration range** for dichlorvos: 10^−6^–10^−10^ M; **LOD**: 3 × 10^−10^ M; **Anti-interference ability**: inorganic ions and ascorbic acid; **Recovery rate** in cucumber and pear juice samples: 96–104%.	[74]
NiCoFeS-based homogeneous sensor	DPV	**Linear concentration range** for trichlorfon: 12.5 fg mL^−1^–1.25 ng mL^−1^; **LOD**: 9.74 fg mL^−1^; **Anti-interference ability**: inorganic ions, ascorbic acid, glucose, tryptophan and leucine; **Repeatability**: RSD of 1.07% (6 times); **Stability**: more than 95% of initial activity (21 days); **Reproducibility**: RSD of less than 5% (5 electrodes); **Recovery rate** in plum, watermelon, lettuce and cucumber samples: 92.8–107.4%.	[75]
CuInS_2_-GO	LSV	**Linear concentration range** for chlorpyrifos: 0.5–230 ng mL^−1^ and 240–470 ng mL^−1^; **LOD**: 0.023 ng mL^−1^; **Anti-interference ability**: inorganic ions, p-nitrophenol and o-nitrophenol; **Repeatability**: RSD of 3.07% (5 times); **Reproducibility**: up to 90.8% (5 electrodes); **Recovery rate** in real samples: 96–106%.	[76]
3D CoXO	DPV	**Linear concentration range** for OPs: 3.88 × 10^−6^–3.88 μM (diazinon) and 4.69 × 10^−7^–0.469 μM (omethoate); **LOD**: 0.36 pM (diazinon) and 0.033 pM (omethoate); **Anti-interference ability**: inorganic ions, glucose, urea, citric acid, chlorpyrifos, methamidophos and carbaryl; **Repeatability**: RSD of 2.98% (5 times); **Stability**: 90.3% of initial activity (30 days, 4 °C); **Reproducibility**: RSD of 2.53% (5 electrodes); **Recovery rate** in real samples: 95.4–104.1%.	[77]
PB	Amperometry	**Linear concentration range** for isocarbophos, chlorpyrifos and trichlorfon: 1 × 10^−7^ g mL^−1^–5 × 10^−6^ g mL^−1^; **LOD**: 10^−7^ g mL^−1^; **Reproducibility**: RSD of 5.4% (3 electrodes); **Recovery rate** in flowering cabbage samples: 84.4–117.4% (chlorpyrifos), 77.1–109.6% (isocarbophos) and 79.1–119.3% (trichlorfon).	[78]
PB-CB	chronoamperometry	**Linear concentration range** for dichlorvos in apple and orange samples: 0–50 ppb; **LOD**: 8 nM (in solution) and 40 nM (dry) (apple), 12 nM (in solution) and 24 nM (dry) (orange); **Repeatability**: RSD of less than 10% (4 times); **Recovery rate**: 83–95% (in solution) and 97–106% (dry) (apple), 81–104% (in solution) and 91–115% (dry) (orange).	[79]
MOF-5	CV	**Linear concentration range** for chlorpyrifos: 30 ng L^−1^–600 ng L^−1^; **LOD**: 3 ng L^−1^; **Sensitivity**: 2.04 µA ng^−1^ L cm^−2^; **Anti-interference ability**: inorganic ions, calcium phosphate, aflatoxinB1 and diisopropyl fluorophosphate; **Stability**: 90.0% of initial activity (15 days).	[84]
Chi-Gra-ZIF-8	DPV	**Linear concentration range** for isocarbophos: 1.73–345.7 nM; **LOD**: 0.62 nM; **Anti-interference ability**: inorganic ions, glucose and urea; **Stability**: 98.09% of initial activity (6 days, 4 °C); **Reproducibility**: RSD of 5.05% (5 electrodes); **Recovery rate** in cabbage and tap water samples: 88.1–122.4%.	[86]
Zn-MOFs	DPV	**Linear concentration range** for OPs: 10^−15^–10^−9^ M (glyphosate), 10^−15^–10^−10^ M (trichlorfon), 10^−15^–10^−9^ M (fenitrothion), 10^−15^–10^−11^ M (iprobenfos), 10^−15^–10^−12^ M (acephate) and 10^−15^–10^−9^ M (methyl-parathion); **LOD**: 1.24 × 10^−13^ M(glyphosate), 8.75 × 10^−14^ M(trichlorfon), 4.40 × 10^−14^ M(fenitrothion), 3.36 × 10^−13^ M(iprobenfos), 7.57 × 10^−14^ M(acephate) and 4.31 × 10^−13^ M(methyl-parathion); **LOQ**: 4.13 × 10^−13^ M (glyphosate), 1.92 × 10^−13^ M (trichlorfon), 1.47 × 10^−13^ M (fenitrothion), 1.12 × 10^−13^ M (iprobenfos), 2.25 × 10^−13^ M (acephate) and 1.44 × 10^−13^ M (methyl-parathion); **Anti-interference ability**: inorganic ions, glucose, uric acid, ascorbic acid, cypermethrin, 2,4-d-butyl ester and propoxur; **Recovery rate** in strawberry, cucumber, oilseed rape and tomato samples: 87.1–109.6%.	[87]
Ce-UiO-66-MWCNTs	DPV	**Linear concentration range** for paraoxon: 0.01–150 nM; **LOD**: 0.004 nM; **Anti-interference ability**: inorganic ions, oxalic acid, citric acid, glucose, carbaryl, 4-nitrophenol, carbofuran, malathion and diazinon; **Repeatability**: RSD of 4.3% (5 times); **Stability**: 85% of initial activity (20 days, 4 °C); **Recovery rate** in cabbage and spinach samples: 95–102%.	[90]
COF NFs	DPV	**Linear concentration range** for dichlorvos: 10^−10^–10^−5^ g L^−1^; **LOD**: 1.50 pg·L^−1^; **Anti-interference ability**: inorganic ions, ascorbic acid and dopamine; **Stability**: almost unchanged (30 days); **Reproducibility**: RSD of 4.2% (6 electrodes); **Recovery rate** in real samples: 85.6–136%.	[94]
COFs-MWCNTs	DPV	**Linear concentration range** for malathion: 1 nM–10 μM; **LOD**: 0.5 nM; **Anti-interference ability**: inorganic ions; **Stability**: 82.9% of initial activity (4 °C); **Reproducibility**: RSD of 2.19% (10 electrodes); **Recovery rate** in real samples: 96–101.6% (tap water) and 98–105% (spinach).	[95]
PDDA-AuNPs-COFs	DPV	**Linear concentration range** for methyl parathion: 1.9 × 10^−9^–3.8 × 10^−5^ M; **LOD**: 2.3 × 10^−10^ M; **Anti-interference ability**: inorganic ions, citric acid, ascorbic acid, glucose, urea, acetamiprid and indoxacarb; **Repeatability**: RSD of 2.4% (5 times); **Stability**: 92.0% of initial activity (14 days, 4 °C); **Reproducibility**: RSD of 2.8% (5 electrodes); **Recovery rate** in tomato and strawberry samples: 95.7–107.9%.	[96]
BPC-COFs	DPV	**Linear concentration range** for trichlorfon: 0.2–19 ng mL^−1^; **LOD**: 0.067 ng mL^−1^; **Anti-interference ability**: inorganic ions, nitrophenol, catechol and hydroquinone; **Stability**: 94% of initial activity (30 days, 4 °C); **Reproducibility**: RSD of 3.9% (5 electrodes); **Reactivation**: up to five times (10 min, 5 mM praldoximin chloride solution); **Recovery rate** in schisandra chinensis samples: 96.1–105%.	[97]

## Data Availability

Data will be made available on request.

## References

[1] Zorbas C., Resnick D., Jones E., Suri S., Iruhiriye E., Headey D., Martin W., Vos R., Menon P. (2025). From promises to action: Analyzing global commitments to tackle hunger and food insecurity. Food Policy.

[2] China Pesticide Information Network. http://www.chinapesticide.org.cn/zwb/detail/31197.

[3] Mali H., Shah C., Raghunandan B.H., Prajapati A.S., Patel D.H., Trivedi U., Subramanian R.B. (2023). Organophosphate pesticides an emerging environmental contaminant: Pollution, toxicity, bioremediation progress, and remaining challenges. J. Environ. Sci..

[4] Jokanović M., Oleksak P., Kuca K. (2023). Multiple neurological effects associated with exposure to organophosphorus pesticides in man. Toxicology.

[5] Li L., Zhou S., Jin L., Zhang C., Liu W. (2010). Enantiomeric separation of organophosphorus pesticides by high-performance liquid chromatography, gas chromatography and capillary electrophoresis and their applications to environmental fate and toxicity assays. J. Chromatogr. B.

[6] Liu Y., Gong S., Ye L., Li J., Liu C., Chen D., Fang M., Letcher R.J., Su G. (2021). Organophosphate (OP) diesters and a review of sources, chemical properties, environmental occurrence, adverse effects, and future directions. Environ. Int..

[7] Irkham, Putra C.P., Kharismasari C.Y., Zakiyyah S.N., Rahmawati I., Anggraningrum I.T., Wahyuni W.T., Valenti G., Paolucci F., Hartati Y.W. (2024). Advancements in electrochemiluminescence-based sensors for ultra-sensitive pesticide residue detection. Sens. Bio-Sens. Res..

[8] Xu X., Zhang W., Huang J., Xu H. (2024). Colorimetric sensors for detection of organophosphorus pesticides in food: From sensing strategies to chemometrics driven discrimination. Trends Food Sci. Technol..

[9] Song K., Zhang L., Meng Y., Ding J., Li X., Liu Y., Ma H. (2026). Recent development in fluorescent probes for monitoring organophosphorus pollutants. Chin. Chem. Lett..

[10] Gupta A., Sharma K., Pandey G., Lawaniya S.D., Pandey H., Awasthi K.K., Awasthi K., Awasthi A. (2025). Combatting organophosphate toxicity: Novel strategies for detection, analysis, and sensor development for public health protection. TrAC Trends Anal. Chem..

[11] Dube A., Malode S.J., Ali Alshehri M., Shetti N.P. (2025). Recent advances in the development of electrochemical sensors for detecting pesticides. J. Ind. Eng. Chem..

[12] Kumaravel A., Chandrasekaran M. (2015). Electrochemical determination of chlorpyrifos on a nano-TiO_2_/cellulose acetate composite modified glassy carbon electrode. J. Agric. Food Chem..

[13] Zhang Q., Yu J., Wei Y., Yang R., Liu J., Su Y., Gao D., Zeng J. (2025). Advances in acetylcholinesterase-based biosensing technologies for organophosphorus pesticide detection: A comprehensive review (2020–2024). Food Chem..

[14] Eto M. (2003). Organophosphorus Insecticides. Encyclopedia of Agrochemicals.

[15] Cheng Y., Lv Y., Zhao X., Lu Y., Jiao T., Ma T., Fu Y. (2025). Multidimensional toxicity of organophosphate pesticides and mitigation strategies for agricultural sustainability. J. Agric. Food Chem..

[16] Karaboga S., Severac F., Collins E.-M.S., Stab A., Davis A., Souchet M., Hervé G. (2024). Organophosphate toxicity patterns: A new approach for assessing organophosphate neurotoxicity. J. Hazard. Mater..

[17] National Center for Biotechnology Information https://pubchem.ncbi.nlm.nih.gov/#query=acephate.

[18] Zinovicius A., Balciunas E., Rozene J., Petroniene J.J., Bogusevice A., Ino K., Striska L., Mockaitis T., Morkvenaite I. (2025). Composites-based electrodes in enzymatic electrochemical glucose biosensors. J. Electroanal. Chem..

[19] Huang L., Qu L., Jia S., Ding S., Zhao J., Li F. (2022). The interaction of allicin with bovine serum albumin and its influence on the structure of protein. Process Biochem..

[20] Tsauria Q.D., Gareso P.L., Tahir D. (2025). Structure–property–application framework for multifunctional chitosan across biomedical, environmental, and technological domains: A review. Int. J. Biol. Macromol..

[21] Strauss J.D., Wagenknecht T. (2013). Structure of glutaraldehyde cross-linked ryanodine receptor. J. Struct. Biol..

[22] Ke Y., Yuan W., Zhou F., Guo W., Li J., Zhuang Z., Su X., Lu B., Zhao Y., Tang Y. (2021). A critical review on surface-pattern engineering of nafion membrane for fuel cell applications. Renew. Sustain. Energy Rev..

[23] Sussman J.L., Harel M., Frolow F., Oefner C., Goldman A., Toker L., Silman I. (1991). Atomic structure of acetylcholinesterase from torpedo californica: A prototypic acetylcholine-binding protein. Science.

[24] Bagrowska W., Karasewicz A., Góra A. (2024). Comprehensive analysis of acetylcholinesterase inhibitor and reactivator complexes: Implications for drug design and antidote development. Drug Discov. Today.

[25] Nicolet Y., Lockridge O., Masson P., Fontecilla-Camps J.C., Nachon F. (2003). Crystal structure of human butyrylcholinesterase and of its complexes with substrate and products. J. Biol. Chem..

[26] Kwak Y., Lee S.-E., Shin J.-H. (2014). Expression of organophosphorus hydrolase in *Escherichia coli* for use as whole-cell biocatalyst. J. Mol. Catal. B Enzym..

[27] Kumaran A., Vashishth R., Singh S., U S., James A., Velayudhaperumal Chellam P. (2022). Biosensors for detection of organophosphate pesticides: Current technologies and future directives. Microchem. J..

[28] Patel H., Rawtani D., Agrawal Y.K. (2019). A newly emerging trend of chitosan-based sensing platform for the organophosphate pesticide detection using Acetylcholinesterase—A review. Trends Food Sci. Technol..

[29] Thakkar J.B., Gupta S., Prabha C.R. (2019). Acetylcholine esterase enzyme doped multiwalled carbon nanotubes for the detection of organophosphorus pesticide using cyclic voltammetry. Int. J. Biol. Macromol..

[30] Ayanda O.S., Mmuoegbulam A.O., Okezie O., Durumin Iya N.I., Mohammed S.E., James P.H., Muhammad A.B., Unimke A.A., Alim S.A., Yahaya S.M. (2024). Recent progress in carbon-based nanomaterials: Critical review. J. Nanopart. Res..

[31] Xie X., Zhou B., Zhang Y., Zhao G., Zhao B. (2021). A multi-residue electrochemical biosensor based on graphene/chitosan/parathion for sensitive organophosphorus pesticides detection. Chem. Phys. Lett..

[32] Kumar T.H.V., Sundramoorthy A.K. (2019). Electrochemical biosensor for methyl parathion based on single-walled carbon nanotube/glutaraldehyde crosslinked acetylcholinesterase-wrapped bovine serum albumin nanocomposites. Anal. Chim. Acta.

[33] Li Y.P., Zhao R.X., Han G.Y., Xiao Y.M. (2018). Novel acetylcholinesterase biosensor for detection of paraoxon based on holey graphene oxide modified glass carbon electrode. Electroanalysis.

[34] Hou W., Zhang Q., Dong H., Li F., Zhang Y., Guo Y., Sun X. (2019). Acetylcholinesterase biosensor modified with ATO/OMC for detecting organophosphorus pesticides. New J. Chem..

[35] Soulis D., Trigazi M., Tsekenis G., Chandrinou C., Klinakis A., Zergioti I. (2020). Facile and low-cost SPE modification towards ultra-sensitive organophosphorus and carbamate pesticide detection in olive oil. Molecules.

[36] Wei M., Feng S. (2017). Amperometric determination of organophosphate pesticides using a acetylcholinesterase based biosensor made from nitrogen-doped porous carbon deposited on a boron-doped diamond electrode. Microchim. Acta.

[37] Rahman M.A., Kumar P., Park D.-S., Shim Y.-B. (2008). Electrochemical sensors based on organic conjugated polymers. Sensors.

[38] Paneru S., Kumar D. (2023). Ag-doped-CuO nanoparticles supported polyaniline (PANI) based novel electrochemical sensor for sensitive detection of paraoxon-ethyl in three real samples. Sens. Actuators B Chem..

[39] Virji S., Huang J., Kaner R.B., Weiller B.H. (2004). Polyaniline nanofiber gas sensors: Examination of response mechanisms. Nano Lett..

[40] Maanaki H., Xu T., Chen G., Du X., Wang J. (2023). Development of integrated smartphone/resistive biosensor for on-site rapid environmental monitoring of organophosphate pesticides in food and water. Biosens. Bioelectron. X.

[41] Jia L., Zhou Y., Wu K., Feng Q., Wang C., He P. (2020). Acetylcholinesterase modified AuNPs-MoS_2_-rGO/PI flexible film biosensor: Towards efficient fabrication and application in paraoxon detection. Bioelectrochemistry.

[42] Holade Y., Hickey D.P., Minteer S.D. (2016). Halide-regulated growth of electrocatalytic metal nanoparticles directly onto a carbon paper electrode. J. Mater. Chem. A.

[43] Loguercio L.F., Griep J., Demingos P.G., Morawski R., Brolo A.G., Andrade G.F.S., Santos J.F.L. (2025). Enhanced enzymatic electrochemical detection of an organophosphate Pesticide: Achieving Wide linearity and femtomolar detection via gold nanoparticles growth within polypyrrole films. Talanta.

[44] Loguercio L.F., Thesing A., Demingos P., de Albuquerque C.D.L., Rodrigues R.S.B., Brolo A.G., Santos J.F.L. (2021). Efficient acetylcholinesterase immobilization for improved electrochemical performance in polypyrrole nanocomposite-based biosensors for carbaryl pesticide. Sens. Actuators B Chem..

[45] Theansun W., Sriprachuabwong C., Chuenchom L., Prajongtat P., Techasakul S., Tuantranont A., Dechtrirat D. (2023). Acetylcholinesterase modified inkjet-printed graphene/gold nanoparticle/poly(3,4-ethylenedioxythiophene):poly(styrenesulfonate) hybrid electrode for ultrasensitive chlorpyrifos detection. Bioelectrochemistry.

[46] Zhang P., Sun T., Rong S., Zeng D., Yu H., Zhang Z., Chang D., Pan H. (2019). A sensitive amperometric AChE-biosensor for organophosphate pesticides detection based on conjugated polymer and Ag-rGO-NH_2_ nanocomposite. Bioelectrochemistry.

[47] Arivazhagan M., Mohan B., Jakmunee J. (2024). Nanostructured metallic enzymes mimic for electrochemical biosensing of glucose. Green Anal. Chem..

[48] Dhull V. (2020). A Nafion/AChE-cSWCNT/MWCNT/Au-based amperometric biosensor for the determination of organophosphorous compounds. Environ. Technol..

[49] Hua Q.T., Ruecha N., Hiruta Y., Citterio D. (2019). Disposable electrochemical biosensor based on surface-modified screen-printed electrodes for organophosphorus pesticide analysis. Anal. Methods.

[50] Bao J., Hou C., Chen M., Li J., Huo D., Yang M., Luo X., Lei Y. (2015). Plant esterase–chitosan/gold nanoparticles–graphene nanosheet composite-based biosensor for the ultrasensitive detection of organophosphate pesticides. J. Agric. Food Chem..

[51] Yao Y., Wang G., Chu G., An X., Guo Y., Sun X. (2019). The development of a novel biosensor based on gold nanocages/graphene oxide–chitosan modified acetylcholinesterase for organophosphorus pesticide detection. New J. Chem..

[52] Cui H.-F., Zhang T.-T., Lv Q.-Y., Song X., Zhai X.-J., Wang G.-G. (2019). An acetylcholinesterase biosensor based on doping Au nanorod@SiO_2_ nanoparticles into TiO_2_-chitosan hydrogel for detection of organophosphate pesticides. Biosens. Bioelectron..

[53] Liu Z., Xia X., Zhou G., Ge L., Li F. (2020). Acetylcholinesterase-catalyzed silver deposition for ultrasensitive electrochemical biosensing of organophosphorus pesticides. Analyst.

[54] Zhang J., Wang B., Li Y., Shu W., Hu H., Yang L. (2019). An acetylcholinesterase biosensor with high stability and sensitivity based on silver nanowire–graphene–TiO_2_ for the detection of organophosphate pesticides. RSC Adv..

[55] Song D., Wang Y., Lu X., Gao Y., Li Y., Gao F. (2018). Ag nanoparticles-decorated nitrogen-fluorine co-doped monolayer MoS_2_ nanosheet for highly sensitive electrochemical sensing of organophosphorus pesticides. Sens. Actuators B Chem..

[56] Yang Y., Zhao Y., Sun F., You T., Gao Y., Yin P. (2020). Electrochemically synthesized superhydrophilic 3D tree-like Ag microstructure for ultrasensitive detection of omethoate. Microchem. J..

[57] Suwannachat J., Saenchoopa A., Tun W.S.T., Patramanon R., Daduang S., Daduang J., Kulchat S. (2024). An electrochemical AChE-based biosensor for organophosphate pesticides using a modified CuNWs/rGO nanocomposite on a screen-printed carbon electrode. Food Chem..

[58] Lu X., Tao L., Li Y., Huang H., Gao F. (2019). A highly sensitive electrochemical platform based on the bimetallic Pd@Au nanowires network for organophosphorus pesticides detection. Sens. Actuators B Chem..

[59] Zhao F., Yao Y., Jiang C., Shao Y., Barceló D., Ying Y., Ping J. (2020). Self-reduction bimetallic nanoparticles on ultrathin MXene nanosheets as functional platform for pesticide sensing. J. Hazard. Mater..

[60] Wu Y., Jiao L., Xu W., Gu W., Zhu C., Du D., Lin Y. (2019). Polydopamine-capped bimetallic AuPt hydrogels enable robust biosensor for organophosphorus pesticide detection. Small.

[61] Yang Y., Yu D., Xu X., Zhao Y., Mi Y., Gao F. (2022). One-dimensional bimetallic PdRh alloy mesoporous nanotubes constructed for ultra-sensitive detection of carbamate pesticide. Anal. Biochem..

[62] Jin R., Kong D., Zhao X., Li H., Yan X., Liu F., Sun P., Du D., Lin Y., Lu G. (2019). Tandem catalysis driven by enzymes directed hybrid nanoflowers for on-site ultrasensitive detection of organophosphorus pesticide. Biosens. Bioelectron..

[63] Chen J., Guo Z., Xin Y., Gu Z., Zhang L., Guo X. (2023). Effective remediation and decontamination of organophosphorus compounds using enzymes: From rational design to potential applications. Sci. Total Environ..

[64] Zhao C., Zhao Y., Wang L., Lu K., Guo W., Lu X., Gao F. (2023). AuNPs and CNTs embellished three-dimensional bloom-like α-Fe_2_O_3_ nanocomposites for highly sensitive electrochemical pesticides detection. Microchem. J..

[65] Luo R., Feng Z., Shen G., Xiu Y., Zhou Y., Niu X., Wang H. (2018). Acetylcholinesterase biosensor based on mesoporous hollow carbon spheres/core-shell magnetic nanoparticles-modified electrode for the detection of organophosphorus pesticides. Sensors.

[66] Yin Z.F., Wu L., Yang H.G., Su Y.H. (2013). Recent progress in biomedical applications of titanium dioxide. Phys. Chem. Chem. Phys..

[67] Bao J., Hou C., Huo D., Dong Q., Ma X., Sun X., Yang M., Galil K.H.A.E., Chen W., Lei Y. (2017). Sensitive and selective electrochemical biosensor based on ELP-OPH/BSA/TiO_2_NFs/AuNPs for determination of organophosphate pesticides with p-nitrophenyl substituent. J. Electrochem. Soc..

[68] Tao S., Guo Y., Wang S., Xu F., Zhou X., Guo Q. (2022). A sensitive and stable acetylcholinesterase biosensor with TiO_2_ nanoparticles anchored on graphitic carbon nanofibers for determination of organophosphate pesticides. Anal. Methods.

[69] Hu H., Wang B., Li Y., Wang P., Yang L. (2020). Acetylcholinesterase sensor with patterned structure for detecting organophosphorus pesticides based on titanium dioxide sol-gel carrier. Electroanalysis.

[70] Cui H.-F., Wu W.-W., Li M.-M., Song X., Lv Y., Zhang T.-T. (2018). A highly stable acetylcholinesterase biosensor based on chitosan-TiO_2_-graphene nanocomposites for detection of organophosphate pesticides. Biosens. Bioelectron..

[71] Liu Y., Xiao Y., Zhang Y., Gao X., Wang H., Niu B., Li W. (2024). ZnO-rGO-based electrochemical biosensor for the detection of organophosphorus pesticides. Bioelectrochemistry.

[72] Li Z., Lu X., Liu G., Yang L., Gao F. (2024). Core-shell ZnO@CoO nitrogen doped nano-composites as highly sensitive electrochemical sensor for organophosphate pesticides detection. Anal. Biochem..

[73] Bao J., Huang T., Wang Z., Yang H., Geng X., Xu G., Samalo M., Sakinati M., Huo D., Hou C. (2019). 3D graphene/copper oxide nano-flowers based acetylcholinesterase biosensor for sensitive detection of organophosphate pesticides. Sens. Actuators B Chem..

[74] Sun Y., Xiong P., Tang J., Zeng Z., Tang D. (2020). Ultrasensitive split-type electrochemical sensing platform for sensitive determination of organophosphorus pesticides based on MnO_2_ nanoflower-electron mediator as a signal transduction system. Anal. Bioanal. Chem..

[75] Wang R., Li B., Li G., Shen Q., Zou L. (2025). NiCoFeS/rGO nanozyme-mediated multifunctional homogeneous sensing system for ultrasensitive electrochemical assay of pesticides residues in fruits and vegetables. Sens. Actuators B Chem..

[76] Itsoponpan T., Thanachayanont C., Hasin P. (2021). Sponge-like CuInS_2_ microspheres on reduced graphene oxide as an electrocatalyst to construct an immobilized acetylcholinesterase electrochemical biosensor for chlorpyrifos detection in vegetables. Sens. Actuators B Chem..

[77] Zhao Y., Li X., Chen J., Lu X., Yang Y., Song D., Gao F. (2022). Porous hierarchical peony-like cobalt-based bimetallic oxides structured by ultrathin nanosheets for highly sensitive electrochemical pesticides detection. Sens. Actuators B Chem..

[78] Shi Q., Teng Y., Zhang Y., Liu W. (2018). Rapid detection of organophosphorus pesticide residue on Prussian blue modified dual-channel screen-printed electrodes combing with portable potentiostat. Chin. Chem. Lett..

[79] Miglione A., Raucci A., Mancini M., Gioia V., Frugis A., Cinti S. (2025). An electrochemical biosensor for on-glove application: Organophosphorus pesticide detection directly on fruit peels. Talanta.

[80] Yu H., Lin P., Zhou L. (2025). Applications and advances of MOFs-based sensors for pesticide and veterinary drug residue detection: From design to application and challenge. J. Food Compos. Anal..

[81] Castelvecchi D., Naddaf M. (2025). Chemistry Nobel for scientists who developed massively porous ‘super sponge’ materials. Nature.

[82] Hoskins B.F., Robson R. (1989). Infinite polymeric frameworks consisting of three dimensionally linked rod-like segments. J. Am. Chem. Soc..

[83] Yaghi O.M., Li G., Li H. (1995). Selective binding and removal of guests in a microporous metal–organic framework. Nature.

[84] Chansi, R. P.R., Mukherjee I., Basu T., Bharadwaj L.M. (2020). Metal organic framework steered electrosynthesis of anisotropic gold nanorods for specific sensing of organophosphate pesticides in vegetables collected from the field. Nanoscale.

[85] Liu F., Liang Z., Zhang Y., Qin S., Zhang S., Zhang T., Li B., Liao X., Li X., Yang S. (2025). Evaluation of the potential application of RAPA-based delivery: Structural evolution and biocompatibility of monodispersed nano-ZIF-8@RAPA. Mater. Today Commun..

[86] Wen L., Wang N., Liu Z., Tao C.-A., Zou X., Wang F., Wang J. (2022). Acetylcholinesterase immobilization on ZIF-8/graphene composite engenders high sensitivity electrochemical sensing for organophosphorus pesticides. Chemosensors.

[87] Wu Q., Wang Y., Wang L., Su Y., He G., Chen X., Hou L., Zhang W., Wang Y.-Y. (2024). A portable electrochemical biosensor based on an amino-modified ionic metal–organic framework for the one-site detection of multiple organophosphorus pesticides. ACS Appl. Mater. Interfaces.

[88] Xue Y., Gao R., Lin S., Zhong Q., Zhang Q., Hong J. (2024). Regulating the interface electron distribution of iron-based MOFs through ligand functionalization enables efficient peroxymonosulfate utilization and catalytic performance. J. Colloid Interface Sci..

[89] Gokila N., Haldorai Y., Saravanan P., Rajendra Kumar R.T. (2024). Non-enzymatic electrochemical impedance sensor for selective detection of electro-inactive organophosphate pesticides using Zr-MOF/ZrO_2_/MWCNT ternary composite. Environ. Res..

[90] Mahmoudi E., Fakhri H., Hajian A., Afkhami A., Bagheri H. (2019). High-performance electrochemical enzyme sensor for organophosphate pesticide detection using modified metal-organic framework sensing platforms. Bioelectrochemistry.

[91] Hosseini M., Sadat Sabet F., Khabbaz H., Aghazadeh M., Mizani F., Ganjali M.R. (2017). Enhancement of the peroxidase-like activity of cerium-doped ferrite nanoparticles for colorimetric detection of H_2_O_2_ and glucose. Anal. Methods.

[92] Abuzeid H.R., El-Mahdy A.F.M., Kuo S.-W. (2021). Covalent organic frameworks: Design principles, synthetic strategies, and diverse applications. Giant.

[93] Song Y., Chen J., Sun M., Gong C., Shen Y., Song Y., Wang L. (2016). A simple electrochemical biosensor based on AuNPs/MPS/Au electrode sensing layer for monitoring carbamate pesticides in real samples. J. Hazard. Mater..

[94] Wang L., Wu N., Wang L., Song Y., Ma G. (2022). Accurate detection of organophosphorus pesticides based on covalent organic framework nanofiber with a turn-on strategy. Sens. Actuators B Chem..

[95] Wang X., Yang S., Shan J., Bai X. (2022). Novel electrochemical acetylcholinesterase biosensor based on core-shell covalent organic framework@multi-walled carbon nanotubes (COF@MWCNTs) composite for detection of malathion. Int. J. Electrochem. Sci..

[96] Yang K., Zhao H., Li N., Wang Y., Sun B., Cui M., Zhang C. (2024). Facile synthesis of novel porphyrin-based covalent organic frameworks integrated with Au nanoparticles for highly sensitive detection of organophosphorus pesticide residues. Microchem. J..

[97] Liu Y., Zhou M., Jin C., Zeng J., Huang C., Song Q., Song Y. (2020). Preparation of a sensor based on biomass porous carbon/covalent-organic frame composites for pesticide residues detection. Front. Chem..

